# Identification of peptides interfering with the LRRK2/PP1 interaction

**DOI:** 10.1371/journal.pone.0237110

**Published:** 2020-08-13

**Authors:** Chang Zhi Dong, Heriberto Bruzzoni-Giovanelli, Yanhua Yu, Karim Dorgham, Christophe Parizot, Jean Marc Zini, Jean Yves Brossas, Pierre Tuffery, Angelita Rebollo

**Affiliations:** 1 Université de Paris, ITODYS, Paris, France; 2 Institute for Interdisciplinary Research, Jianghan University, Wuhan, China; 3 Université de Paris, Centre d’Investigation Clinique Inserm/AP-HP Hôpital Saint Louis, Paris, France; 4 Sorbonne Université, Inserm, Centre d’Immunologie et des Maladies Infectieuses (CIMI-Paris), Paris, France; 5 Sorbonne Université, Inserm, Centre d’immunologie et des Maladies Infectieuses-Paris (Cimi-Paris), Paris, France; 6 AP-HP Hopital Saint Louis, Paris, France; 7 Université de Paris, BFA, Inserm, RPBS, Paris, France; University of Rochester School of Medicine and Dentistry, UNITED STATES

## Abstract

Serine/threonine phosphatases are responsible for modulating the activities of the protein kinases implicated in the development of several pathologies. Here we identified by a PEP-scan approach a peptide of LRRK2, a Parkinson’s disease associated protein, interacting with the phosphatase PP1. In order to study its biological activity, the peptide was fused via its N-terminal to an optimized cell penetrating peptide. We synthesized from the original peptide five interfering peptides and identified two (Mut3DPT-LRRK2-Short and Mut3DPT-LRRK2-Long) able to disrupt the LRRK2/PP1 interaction by competition in anti-LRRK2 immunoprecipitates. Using FITC-labelled peptides, we confirmed their internalization into cell lines as well as into primary cells obtained from healthy or ill human donors. We confirmed by ELISA test the association of Mut3DPT-LRRK2-Long peptide to purified PP1 protein. The peptides Mut3DPT-LRRK2-5 to 8 with either N or C-terminal deletions were not able to disrupt the association LRRK2/PP1 nor to associate with purified PP1 protein. The interfering sequences blocking the PP1/LRRK2 interaction were also fused to a shuttle peptide able to cross the blood brain barrier and showed that the newly generated peptides BBB-LRRK2-Short and BBB-LRRK2-Long were highly resistant to protease degradation. Furthermore, they blocked PP1/LRRK2 interaction and they penetrated into cells. Hence, these newly generated peptides can be employed as new tools in the investigation of the role of the LRRK2/PP1 interaction in normal and pathological conditions.

## Introduction

Serine/threonine protein phosphatases 1 (PP1) and phosphatase 2A (PP2A) are the most widely distributed and abundant serine/threonine phosphatases in eukaryotic cells. They are involved in the regulation of several essential cellular functions such as proliferation, apoptosis, memory etc[1, 2]. In vertebrates, nearly 200 molecules have been validated as partners of PP1. The binding of PP1 to PP1 Interacting Proteins (PIPs)is mediated by short sequences, and in most cases, these short linear sequences combine to form large specific PPI-binding interfaces[3, 4]. Although PIPs are often variants of the corresponding PP1 binding sequence, they are different in the number and combination of docking sites. PIPs regulate the activity of associated PP1 by blocking their interaction with other partners or blocking the access to the active phosphatase site. Many PP1 partners have different domains for their association with and their regulation of PP1 and for substrate recruitment and sub cellular targeting. As a consequence, this allows the direct association of PP1 to a specific substrate. Thus, PP1 acts as a catalytic subunit for a large number of holoenzymes, each with its own substrates and regulation machinery. The variety of the PP1 associations and their characteristics accounts for the specificity of PP1 *in vivo*[5, 6].

Many naturally occurring protein phosphatase inhibitors with different relative PP1/PP2A affinities have been described and are widely used as powerful research tools. In particular microcystins act as highly toxic cyanotoxins by binding to their main targets, PP1 and PP2A[6]. Similarly, low concentrations of okadaic acid have been used to specifically inhibit PP2A while it also inhibits PP1/PP2A when used in higher concentrations[7, 8].

Leucine-Rich Repeat Kinase 2 (LRRK2) is a PP1 interacting protein[9]. LRRK2 gene mutations can lead to familial Parkinson's disease (PD) and are considered to constitute a risk factor of PD even though the underlying molecular mechanism is not understood. A cluster of phosphorylation sites in LRRK2[10–13], including the Serine 910, 935, 955 and 973, contribute to PD pathology, since various LRRK2 mutants linked to PD are dephosphorylated at these residues. LRRK2 is dephosphorylated after inhibition of kinase activity, which has been suggested as an approach for PD treatment[14, 15]. In addition, PP1 is also responsible for dephosphorylation of LRRK2 and subsequent inhibition of LRRK2 kinase activity[9, 11].

Protein phosphatases have both protective and promoting roles in several diseases such as tumoral transformation. An example is both pro-tumoral and tumor-suppressing function of protein phosphatases these underscore the importance of identifying phosphatase regulators[8, 16]. In deed, a few regulators of protein phosphatase activity are already in clinical use. However, these were not developed by target-directed approaches. Thus, it is interesting to develop targeted regulators of phosphatases, (particularly those involved in pathological process) by either targeting their enzymatic site or the sites of interaction with their partners, that can serves as research tools. We and others have developed interfering peptides targeting PP2A interactions and showed their potential interest in the development of therapeutic strategies[17–19]. We now present interfering peptides targeting the interaction between PP1 and LRRK2 in order to provide new tools to understand the biological significance of interaction, as well as demonstrating the therapeutic potential of such blocking peptides.

## Materials and methods

### Peptides synthesis and sequence

Fmoc/*t*Bu strategy was selected for the peptide synthesis except for the first C-terminal residue, lysine of which the side chain was protected with dde group. The peptide-anchored resin was handled with 2% monohydrate hydrazine in DMF according to the manufacture recommended method to remove the dde group. The peptide-anchored resin was then shaken in DMF with either FITC-NCS alone (1.5 eq.) or biotin (3 eq.) in the presence of DCC (3 eq.)/HOBt (3 eq.)/DIEA (5 eq.) overnight at room temperature. After washing 4 times with DMF and 4 times with CH_2_Cl_2_, the peptide was finally cleaved from the resin and precipitated twice with cold ether/heptane (1/1). It was then dissolved in 30% CH_3_CN in water and lyophilized. The purification was performed by RP-HPLC using an increasing CH_3_CN gradient. Its identity was confirmed by MALDI-mass spectrometry (Bruker). The peptides are patent pending (PCT/EP2020/057898, Unversite de Paris)

### Peptide structure modelling

The structure of the long sequence was predicted using PEP-FOLD3[20] in house implementation and subject to visual inspection to identify candidate N- and C-terminal amino acids deletions likely to question peptide interfering ability.

### Cell lines

Human cancer breast cell line MDA-MB231 (ATCC HTB-26) was cultured in DMEM medium supplemented with 10% foetal calf serum (FCS, Thermo Fischer Scientific). Peripheral blood mononuclear cells (PBMC) were cultured in RPMI medium (Gibco) supplemented with 10% of FCS.

### PP1 binding assay on cellulose-bound peptides containing LRRK2 sequence (PEP-scan)

Overlapping dodecapeptides with two amino acid shift, spanning the complete LRRK2 sequence were prepared by automatic spot synthesis (Abimed, Langerfeld, Germany) onto an amino-derived cellulose membrane, as described[21, 22]. The membrane was saturated using 3% non-fat dry milk/3% BSA (Sigma-Aldrich) (2h room temperature), incubated with purified PP1alpha catalytic protein (Sigma P7937, 4 μg/ml, 4°C, overnight) and after several washing steps, incubated with polyclonal anti-PP1 antibody (Santa Cruz, sc7482, 1:500 dilution) 2h at room temperature, followed by HRP-conjugated secondary antibody (DAKO, PO447, 1:1000 dilution) for 1h at room temperature. Positive spots were visualized using the ECL system (Bio-Rad, 170–5060).

### Comparative modelling of the LRRK2 domain

The comparative modelling of the LRRK2 domain was done using the hhsearch suite[23] to identify a possible 3D template of the Protein Data Bank[24] 3D modelling was performed using as template the 3DPT PDB entry and the Tito software[25] to refine the hhsearch alignment, and identify preserved regions of the template. Loops were then built using the DaReUS-loop approach[26].

### Isolation and culture of primary cells

Fresh blood from healthy donors (HD) was obtained from Etablissement Français du Sang. CLL patient samples were obtained from the Department of Hematology of the Hospital Saint Louis upon approval of the project by Ministry of Higher Education and Research (CODECOH DC-2018-3261) and signed informed consent of the patients according to the French law of using human samples. All the methods were performed in accordance with the guidelines and regulations of French law. Peripheral blood mononuclear cells (PBMC) from HD and CLL patients were prepared by Ficoll(Sigma Aldrich) gradient centrifugation, as previously described[17]. Cells were maintained in RPMI 1640 (Gibco) supplemented with 10% of FCS, 1% non-essential amino acids (Gibco), 1% Hepes (Gibco), 1% sodium pyruvate (Gibco) and 1% glutamine (Gibco).

### Immunoprecipitation, western blot and *in vitro* protein/protein interaction competition

The protocol was previously described[18]. Briefly, cells (5x10^6^) were lysed for 20 min at 4°C in lysis buffer (50 mM Tris pH8, 1% NP40, 137 mM NaCl, 1 mM MgCl_2_, 1 mM CaCl_2_, 10% glycerol and protease inhibitor mixture Sigma Aldrich). Lysates (500 μg) were immunoprecipitated with the appropriated antibody overnight at 4°C and protein A/G Sepharose (Santa Cruz) was added for 1h at 4°C. After washing with 1x TBST (20 mM Tris-HCl pH7.5, 150 mM NaCl, 0.05% Tween 20), the PP1/LRRK2 interaction was competed using 1 mM of the Mut3DPT-LRRK2-Long or Mut3DPT-LRRK2-Shor peptide for 30 min at room temperature. After several washing steps, immunoprecipitates were separated by SDS-PAGE, transferred to nitrocellulose and blotted with anti PP1 antibody (Santa Cruz or Thermo Fisher 1:500 dilution). The membrane was washed and incubated with PO-conjugates secondary antibody (Dako, 1:1000 dilution). Protein detection was performed using the ECL system (Bio-Rad). As internal control, the blot was also hybridized with anti-LRRK2 antibody (Abcam, ab133474, 1;500 dilution). Western blots were densitometred using Image J. Statistic analysis were done using Anova.

### Quantification of cellular internalization

Human cell line MDA-MB231 was seeded in 24 well plate (1x10^5^ cells/well) and treated with different concentrations of FITC-labelled peptides (GL-Biochem) or for different periods of time. After treatment, cells were harvested and washed twice with PBS to remove the extracellular unbound peptide and resuspended in 200 μL of PBS. FITC fluorescence intensity of internalized peptides was measured by flow cytometry on a FACSCanto II as previously described[19] (Beckton Dickinson). Data were analysed with FACSDiva 6.1.3 software (DB Biosciences). Untreated cells were used as control. For PMBC from HD or CLL patients, cells were maintained in RPMI culture medium.

### Peptide internalization visualization

For intracellular localization of FITC-labelled peptides, MDA-MB231 cells were seeded in a 8 well Labtek (Thermo Fisher). Cells were treated with FITC-labelled peptides for 4 h and fixed with 4% of formaldehyde for 15 min at room temperature. Samples were washed twice with PBS and mounted in mounting buffer (Thermo Fisher) as described[19]. Images were captured with a fluorescence microscopy (Olympus Japan) using 40x magnification objective.

### Characterization of PP1 and LRRK2 peptide interaction by ELISA

A total of 100 μL of biotinylated peptides diluted at 100 μM in PBS were incubated for 2 h at room temperature in a 96-wel Streptavidine coated plate (Pierce, 15128). Wells were washed five times with PBS/0.05% Tween-20 (PBST) and filled with 100 μL of PP1 (Sigma, P7937) diluted in PBS/2.5% BSA (Sigma Aldrich) at the indicated dilutions. Plate were incubated over night at 4°C and washed five times with PBST. A total of 100 μL of rabbit polyclonal IgG anti-human PP1α (FL-18) (Santa Cruz Biotechnology, sc-7482) were added at 5 μg/mL in PBS/BSA for 1 h at room temperature. Wells were washed 5 times with PBST and filled with 100 μL of HRP conjugated anti-rabbit IgG (Sigma, A-0545) diluted at 1:20,000 in PBS/BSA for 1 h at room temperature. Wells were washed 5 times with PBST and 100 μL of TMB substrate (Pierce, 34021) were added and incubated for 15–45 min. The reaction was stopped with 50 μL of 2 N sulphuric acid, and the absorbance was measured at 450 nm on a Multiskan EX plate reader (Thermo Scientific).

### Analysis of peptide integrity on human serum

Peptides were incubated at 37°C in 250 μl of human serum (Sigma Aldrich) for different periods of time. Samples were collected and peptide degradation stopped by freezing. Peptides were extracted from samples using the Proteo Miner Protein Enrichment System (Bio-Rad). Percentage of intact peptide was estimated by mass spectrometry (MS) using MALDI-TOFF as described previously[19] (Bruker Autoflex II) following their protocol. Measurements were performed in triplicate. MS data were analysed using the software Cliprot tools, Felx analysis, Bruker.

## Results

### Identification of LRRK2 sequences involved in binding to PP1

**Fig 1:**
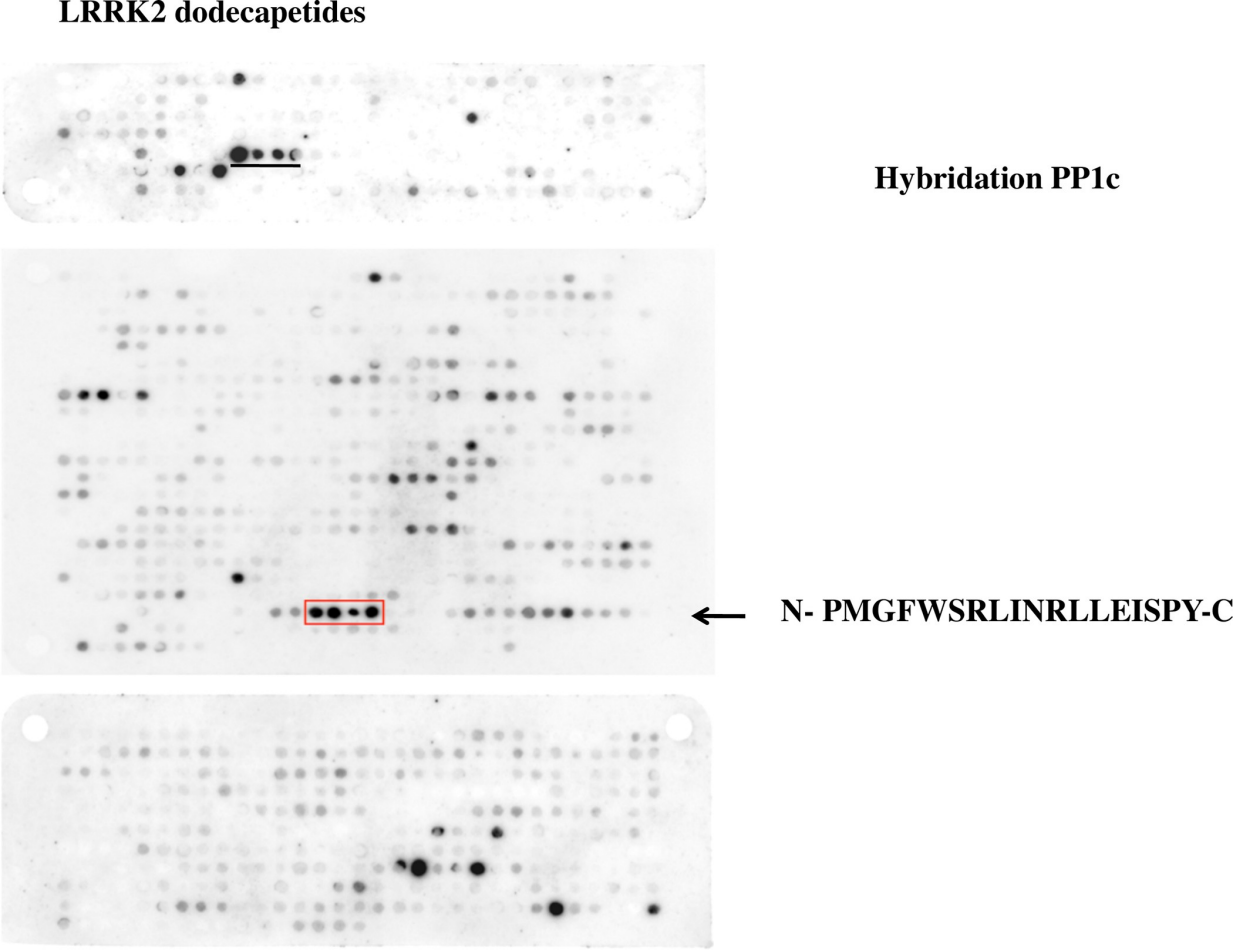
Identification of the binding site of LRRK2 to PP1c. The sequence of LRRK2 was developed as series of overlapping dodecapeptides with a shift of two amino acids. The membrane was hybridized with purified PP1alpha catalytic subunit protein, followed by an anti-PP1 antibody and a secondary antibody. Spots were detected using the ECL system. LRRK2 peptides that interact with PP1c are boxed and the sequence shown (1701–1718). A supplementary sequence is underlined in the upper membrane of the figure.

**Fig 2:**
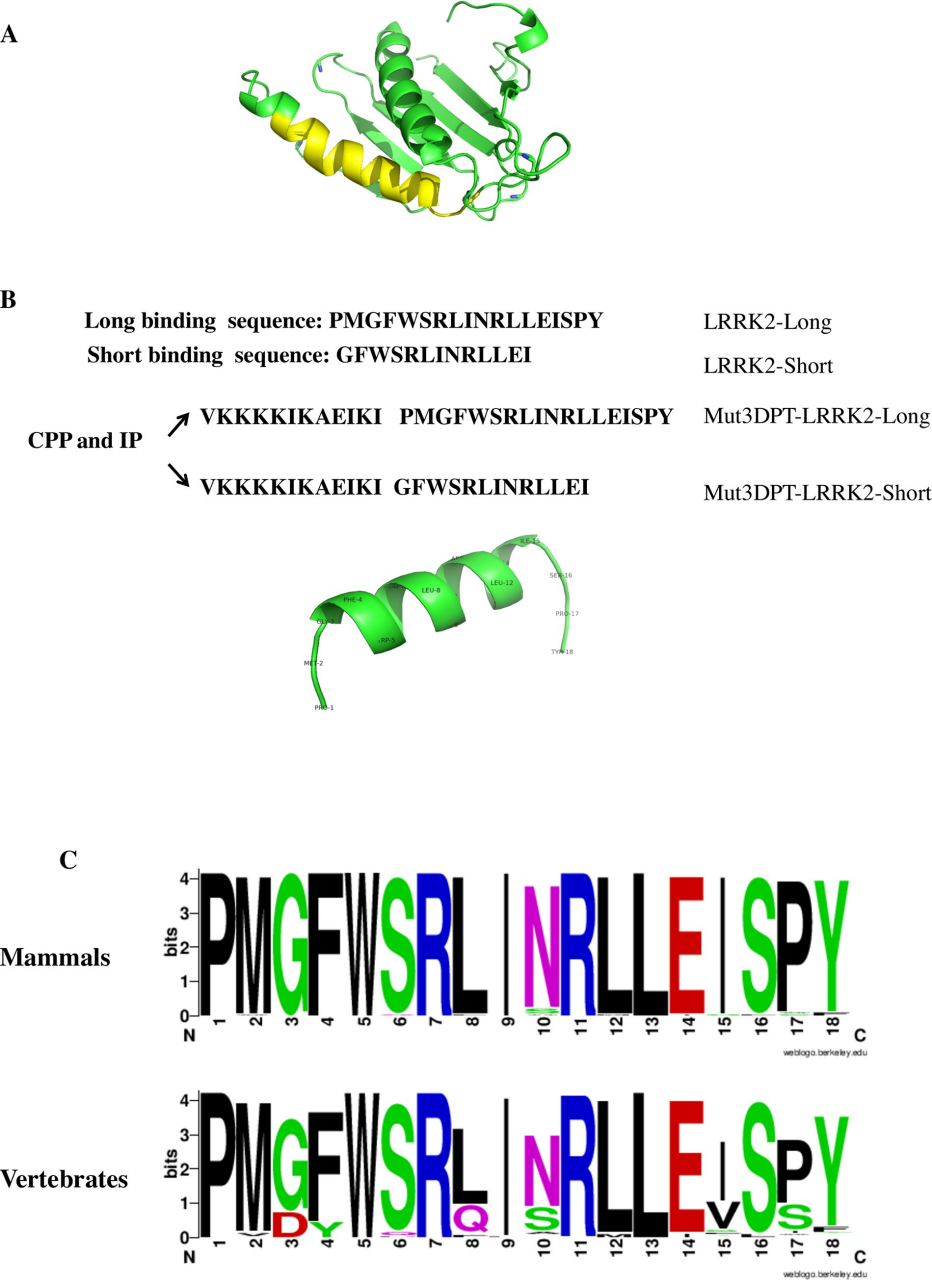
Structure of the Short and Long sequence of the LRRK2 interacting peptides. A) Homology model of the LRRK2 domain encompassing the interfering fragment (depicted in yellow). Image was generated using PEP-FOLD software (https://bioserv.rpbs.univ-paris-diderot.fr/services/PEP-FOLD3/). B) Sequence of the short and long interfering peptides. The sequences were associated to a cell penetrating peptide named Mut3DPT, previously described for generation of the cell penetrating and interfering peptides. The helical structure of the long interfering peptide is also shown, indicating the amino acids that were deleted in the short version of the peptide. Models were generated using the PEP-FOLD3 software (https://bioserv.rpbs.univ-paris-diderot.fr/services/PEP-FOLD3/) and images were generated using pymol 2.1.0 (https://pymol.org). C) Sequence variation observed through related Uniprot mammal (top) and vertebrate (bottom) sequences. Image was generated using weblogoberkely (https://weblogo.berkeley.edu/logo.cgi).

In order to determine which LRRK2 amino acid residues mediate binding to the serine/threonine phosphatase PP1, we employed a PEP-scan approach. Overlapping dodecapeptides covering the whole amino acid sequence of LRRK2 were immobilized on a cellulose membrane and hybridized with the PP1alpha catalytic subunit (Fig 1). A set of 4 contiguous spots revealed the presence of a linear interacting motif spanning the residues 1701 to 1718 of the LRRK2 sequence. The motif is embedded in amino acids 1512 to 1878, a region not annotated in the Uniprot entry Q5S007, corresponding to the COR domain of LRRK2, that previously has been shown to be involved in dimerization. Homology modelling was successful for the region 1688 to 1829, i.e. a domain encompassing the interfering fragment. In addition, a set of 4 spots is also observed in the upper part of the membrane. As depicted in Fig 2A, this LRRK2 region (1701 to 1718) is exposed to solvent, and adopts anα helical conformation, consistant with its ability to mediate binding to PP1. Such helical conformation is also predicted for the peptide in isolation (Fig 2B) by the PEP-FOLD software. A chimeric peptide was synthesized containing the newly identified PP1-interacting sequence of LRRK2 fused to an optimized cell penetrating peptide, Mut3DPT-Sh1 (VKKKKIKAEIKI)m an optimized peptide derived from DPT-Sh1 ([27]. The chimeric peptide, named Mut3DPT-LRRK2-Long, was then used for functional analysis. To minimize the entropic cost upon peptide binding we analysed whether the original interacting sequence could be shortened. As shown on Fig 2B, although the peptide is predicted to adopt a helical conformation, its N- and C-terminal regions appeared less structured. A shorter peptide (Mut3DPT-LRRK2-Short) lacking the first 2 and the last 3 amino acids was designed using PEP-FOLD prediction software. A search for sequence variants in modelled mammalian sequences was performed using the Uniprot server with the proposed default parameters and an e-value less than 0.00001. Over 129 matches were identified, revealing a strong conservation at most positions of the interfering fragment (Fig 2C), 8 out 18 residues being strictly conserved among the vertebrate sequences[28]. The other positions showed only limited variation, suggesting possible functional constraints for this fragment.

### *In vitro* competition of LRRK2/PP1 interaction

**Fig 3:**
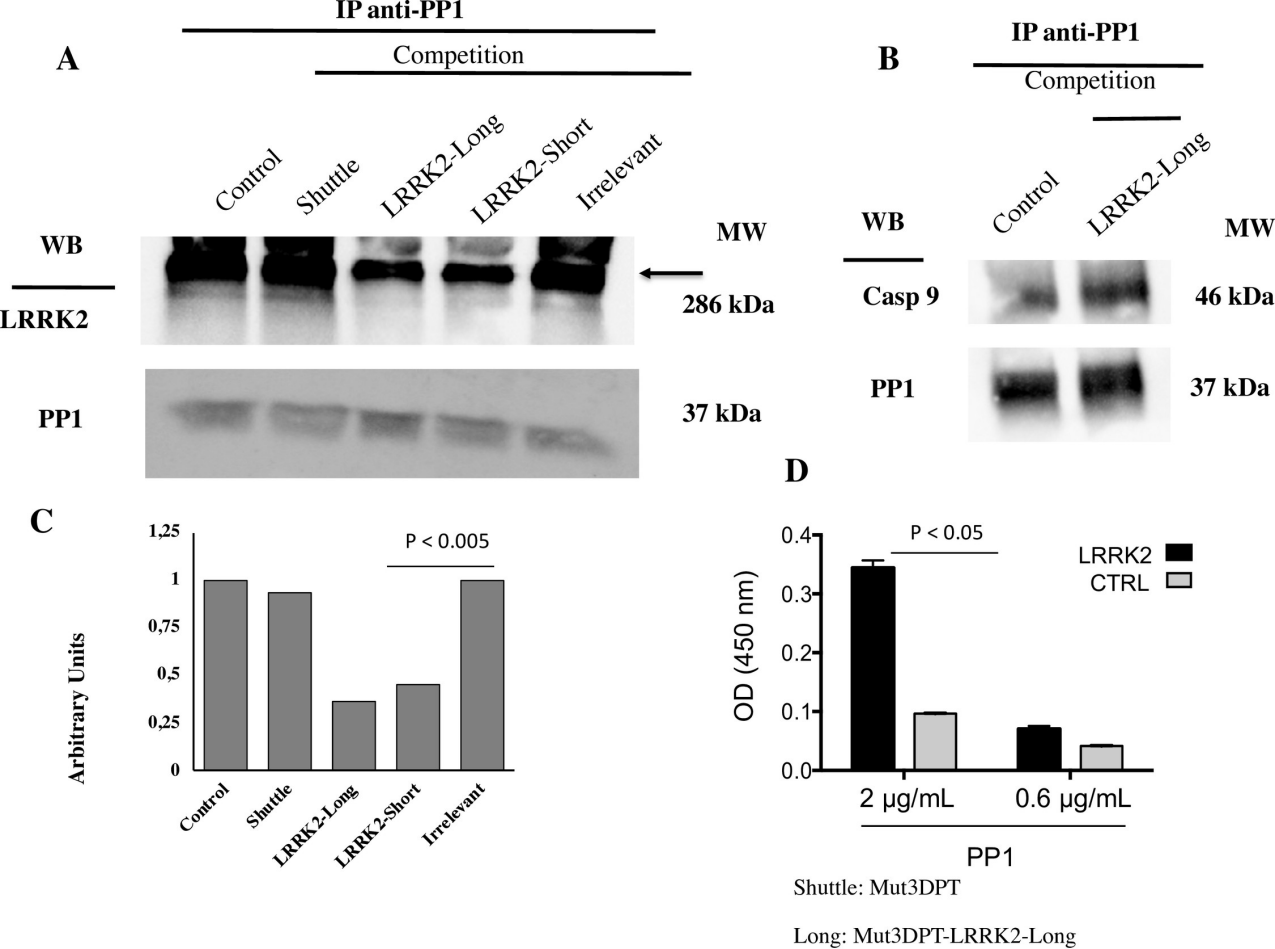
Mut3-DPT-LRRK2-Long peptide competes *in vitro* LRRK2/PP1c interaction. A) MDA-MB231 cells were lysed and cytoplasmic extracts immunoprecipitated with anti-PP1 antibody (Santa Cruz). LRRK2/PP1c interaction was competed *in vitro* with 1 mM of Mut3DPT-LRRK2-Long and Mut3DPT-LRRK2-Short peptides for 30 min at room temperature. Immunoprecipitates were washed and blotted with anti-LRRK2 (Abcam) and anti-PP1 antibody (Santa Cruz or Thermo Fisher), the former as internal control of protein loading. Immunoprecipitates competed with the shuttle alone or an irrelevant peptide were used as control. Data are representative of three independent experiments. B) Cytoplasmic extracts were isolated as above. The PP1/caspase 9 interaction was competed with Mut3DPT-LRRK2-Long peptide for 30 min at room temperature. Blot was treated as above. C) Densitometric analyses of the western blot. The competition with the irrelevant peptide was compared to the competition with LRRK2-Short and LRRK2-Long peptides (p <0.005). D) Mut3DPT-LRRK2-Long and control (CTRL) biotinylated peptides were immobilized on a Streptavidine coated plate and incubated overnight with dilutions of PP1alpha catalytic subunit at 2 and 0.6 μg/mL. After washing, rabbit anti-PP1alpha was added in each well and incubated 1 h at room temperature. Wells were washed and filled with a dilution of HRP-conjugated anti-Rabbit. Binding activity of PP1α is expressed as mean OD at 450 nm of duplicate wells, and bars indicate SD. These data are representative of 2 independent experiments (p <0.05).

An *in vitro* competition assay was performed in order to confirm that Mut3DPT-LRRK2-Long and Mut3DPT-LRRK2-Short peptides target the LRRK2/PP1 interaction. Lysates of the MDA-MB231 cell line were immunoprecipitated with anti-PP1 antibody and the interaction with LRRK2 was competed using Mut3DPT-LRRK2-Long and Mut3DPT-LRRK2-Short peptides (Fig 3A). LRRK2 was detected in the control anti-PP1 immunoprecipitates and in immunoprecipitates competed using the shuttle alone (Mut3DPT) or an irrelevant peptide, whereas the level of detection was much lower after competition with 1 mM of Mut3DPT-LRRK2-Long or Mut3DPT-LRRK2-Short peptide. Amounts precipitated PP1 was used as internal control and showed similar levels in all conditions. To confirm the PP1/LRRK2 targeting of the peptide, the interaction PP1/caspase 9 was competed using Mut3DPT-LRRK2-Long peptide. Fig 3B, shows that the interaction was not affected by the peptide, suggesting that Mut3DPT-LRRK2-Long peptide target the interaction between human LRRK2 and PP1. Fig 3C show the densitometric analyse of bands of the western blot on Fig 3A. We analysed next whether biotinylated Mut3DPT-LRRK2-Long was able to associate with purified PP1 protein. Fig 3D shows that the peptide recognized the protein PP1 in an ELISA test (p<0.05). An irrelevant peptide (biotinylated Min-F8-1 peptide) was used as negative control.

**Fig 4:**
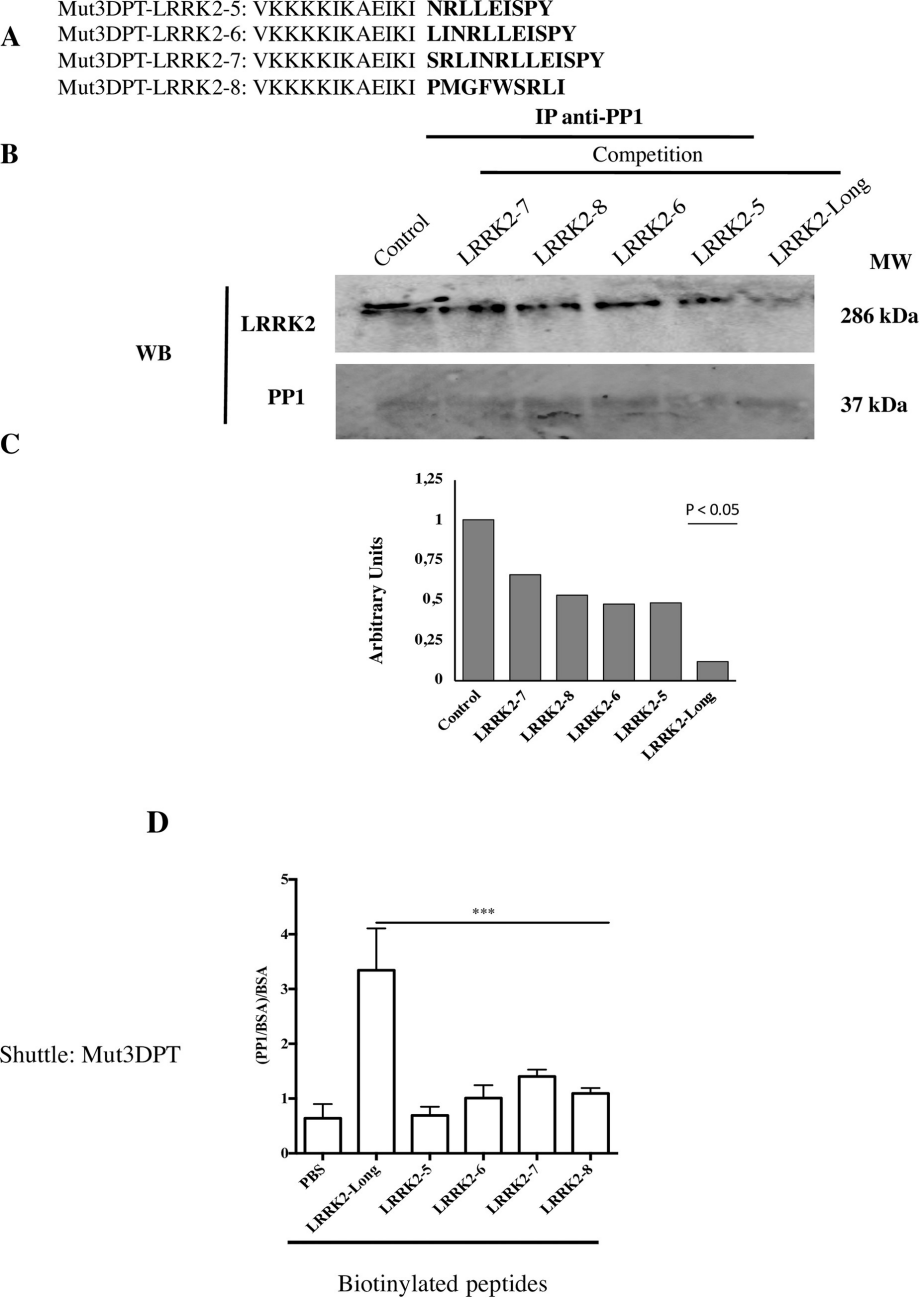
*In vitro* competition of LRRK2/PP1c interaction using LRRK2-5 to LRRK2-8 peptides. A) Sequence of the new generated versions of the Mut3DPT-LRRK2-Long peptide, LRRK2-5 to LRRK2-8. B)MDA-MB231 cell line was lysed and cytoplasmic extracts immunoprecipitated with anti-PP1 antibody. The LRRK2/PP1c interaction was competed *in vitro* with 1 mM of peptides for 30 min at room temperature. Immunoprecipitates were washed and blotted with anti-LRRK2 antibody. Data are representative of two independent experiments. Competition with or without Mut3DPT-LRRK2-Long peptide was used as control. PP1 expression was used as control of loading. C) Densitometric analyses of the western blot showing the comparison of the control to the LRRK2-Long peptide competition for the determination of statistic significance. p <0.05). D) Biotinylated peptides were immobilized on a Strepavidine coated plate and incubated overnight with purified PP1alpha protein. After washing, rabbit anti-PP1α was added in each well and incubated 1 h at room temperature. Wells were washed and filled with a dilution of HRP-conjugated anti-Rabbit. Binding activity of PP1α is expressed as mean OD at 450 nm of triplicate wells, and bars indicate SD. The p value is also shown on the figure.

We investigated whether the shorter versions of the original peptide, Mut3DPT-LRRK2-5 to Mut3DPT-LRRK2-8 (Fig 4A), were able to compete *in vitro* binding PP1 by LRRK2. At a concentration of 1 mM the Mut3DPT-LRRK2-Long peptide strongly ablates the binding PP1/LRRK2 (Fig 4B). The shorter versions of the peptide slightly block PP1/LRRK2 interaction, although with much lower efficacy than Mut3DPT-LRRK2-Long peptide (Fig 4C). We analysed by ELISA test whether the biotinylated versions of Mut3DPT-LRRK2-5 to Mut3DPT-LRRK2-8 were able to associate to purified PP1. Fig 4D shows that these peptides failed to associate with purified PP1 protein, *in vitro*(p<0.001). As positive control, the original Mut3DPT-LRRK2-Long peptide is able to associate to PP1. In summary, Mut3DPT-LRRK2-Long and Mut3DPT-LRRK2-Short recognize PP1 while the other variant shave lost the capacity to recognize PP1.

### Quantification of internalization of Short and Long versions of interfering peptides

**Fig 5:**
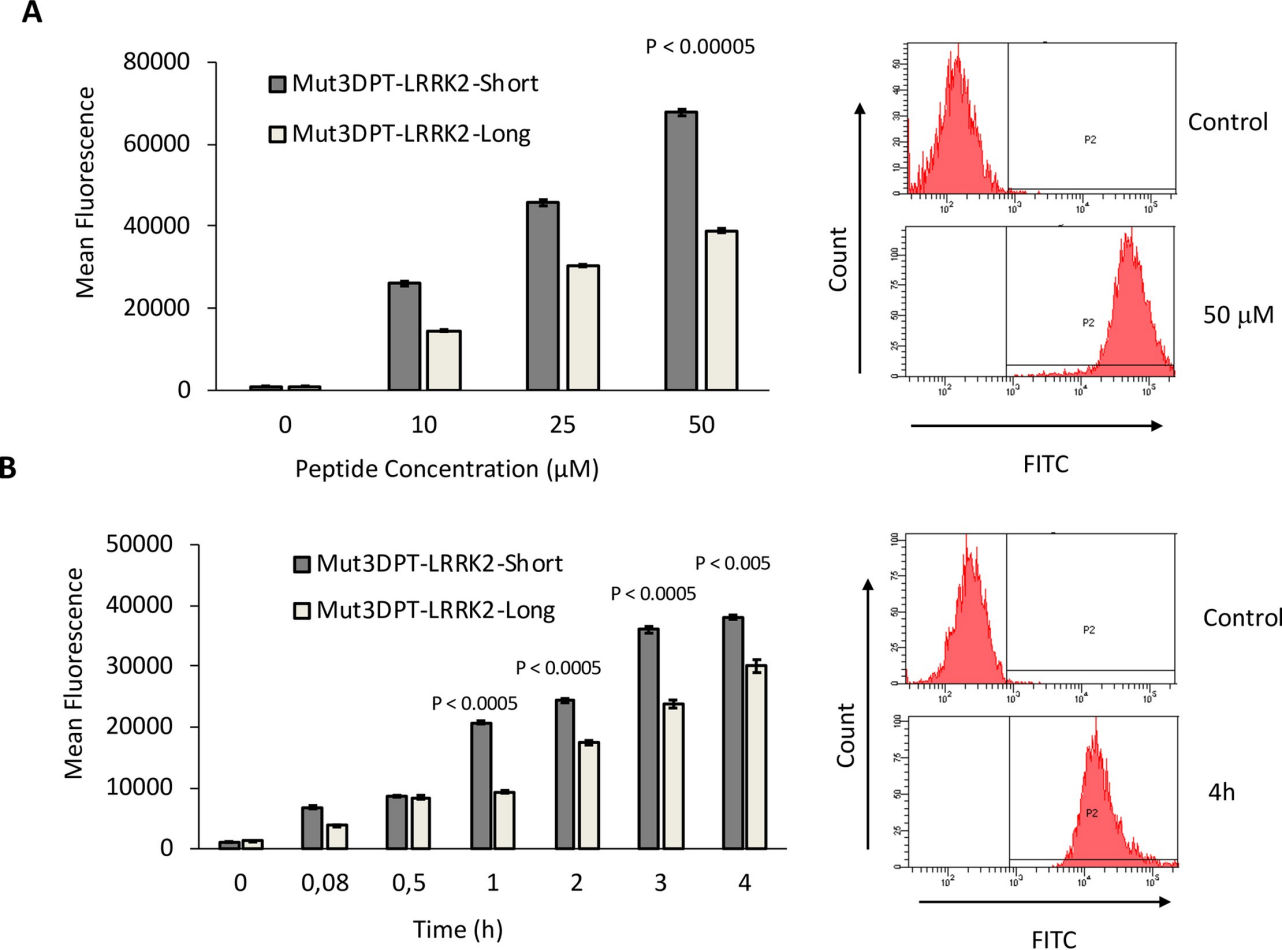
Concentration and time-dependent internalization of FITC-labelled Mut3DPT-LRRK2-Short and Mut3DPT-LRRK2-Long peptides. A) MDA-MB231 cells were incubated 4h with different concentrations of FITC-labelled peptides. The mean fluorescence intensity was detected by flow cytometry (FACS) and compared to non-treated control cells. The experiment was repeated two times with triplicates. Standard deviation is shown, as well as p values. B) MDA-MB231 cells were incubated with 20 μM of the FITC-labelled peptides for different periods of time. The mean fluorescence intensity was detected as above. Non-treated cells were used as control. Bars indicate standard deviation. Data are representative of two experiments with triplicate samples. SD is shown, as well as p values. A representative plot histogram of flow cytometry of control cells, cells treated with 50 μM of peptide or cells treated for 4h with the peptide is shown.

We evaluated whether Mut3DPT-LRRK2-Long and Mut3DPT-LRRK2-Short peptides were able to internalize into cells. The peptides were labelled with FITC and internalization was analysed by FACS. MDA-MB231 cells were exposed for 4h to FITC-labelled Mut3DPT-LRRK2-Long and Mut3DPT-LRRK2-Short peptides at different concentration and internalization analysed by flow cytometry (FACS) (Fig 5A). The fluorescence intensity detected appeared higher for Mut3DPT-LRRK2-Short peptide, compared to Mut3DPT-LRRK2-Long peptide, which showed lower fluorescence intensity (p<0.0005). The influence of time of incubation on internalization was examined ata fixed peptide concentration and again Mut3DPT-LRRK2-Short peptide showed a higher level of internalization (Fig 5B). A representative flow cytometry plot of control, 50 μM concentration and 4h of incubation is shown. Taken together, our results suggest that Mut3DPT-LRRK2-Short and Mut3DPT-LRRK2-Long peptides are internalized into cells.

### Internalization of Mut3DPT-LRRK2-Long and Mut3DPT-LRRK2-Short peptides into primary cells

**Fig 6:**
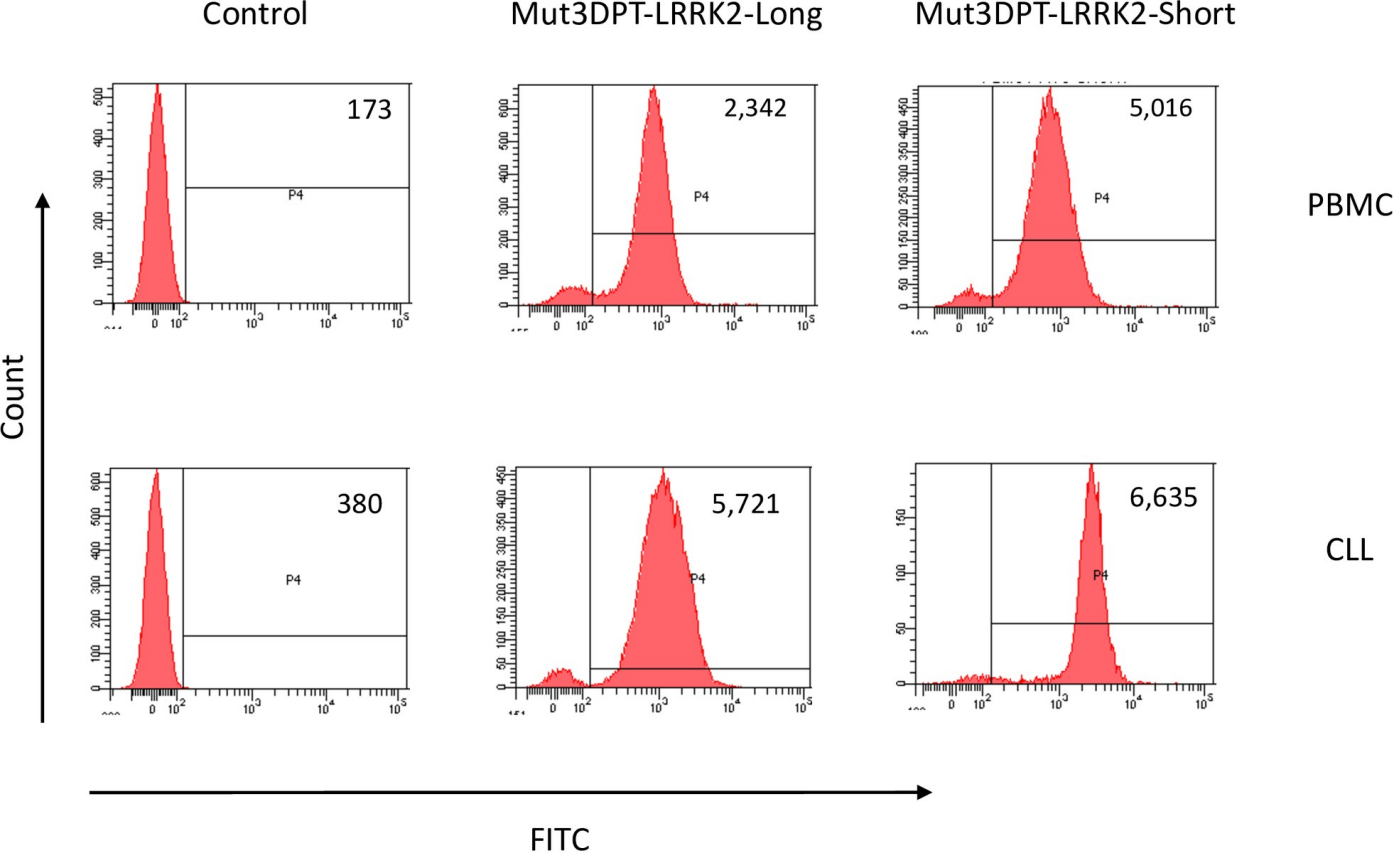
Internalization of FITC-labelled Mut3DPT-LRRK2-Short and Mut3DPT-LRRK2-Long peptides on healthy and tumoral PBMC. PBMC of healthy donors (HD) or CLL patients were incubated 4h with 50 μM of the FITC-labelled Mut3DPT-LRRK2-Short and Mut3DPT-LRRK2-Long peptides. The mean fluorescence intensity was detected by flow cytometry (FACS). The mean fluorescence intensity is shown in each plot histogram. Non-treated cells were used as control. Data are representative of four different patients and healthy donors.

In addition to cell lines, we also tested internalization of the Mut3DPT-LRRK2-Short and Mut3DPT-LRRK2-Long peptides in peripheral blood mononuclear cells (PBMC) obtained from healthy donors or Chronic Lymphocytic Leukemia (CLL) patients. PBMC from both sets of donors were incubated with 50 μM of both peptides for 4h at 37°C. As illustrated in Fig 6, Mut3DPT-LRRK2-Short looks slightly higher fluorescence intensity than Mut3DPT-LRRK2-Long for both, healthy donors and CLL patients.

### Intracellular localization of the Mut3DPT-LRRK2-Short and Mut3DPT-LRRK2-Long peptides

**Fig 7:**
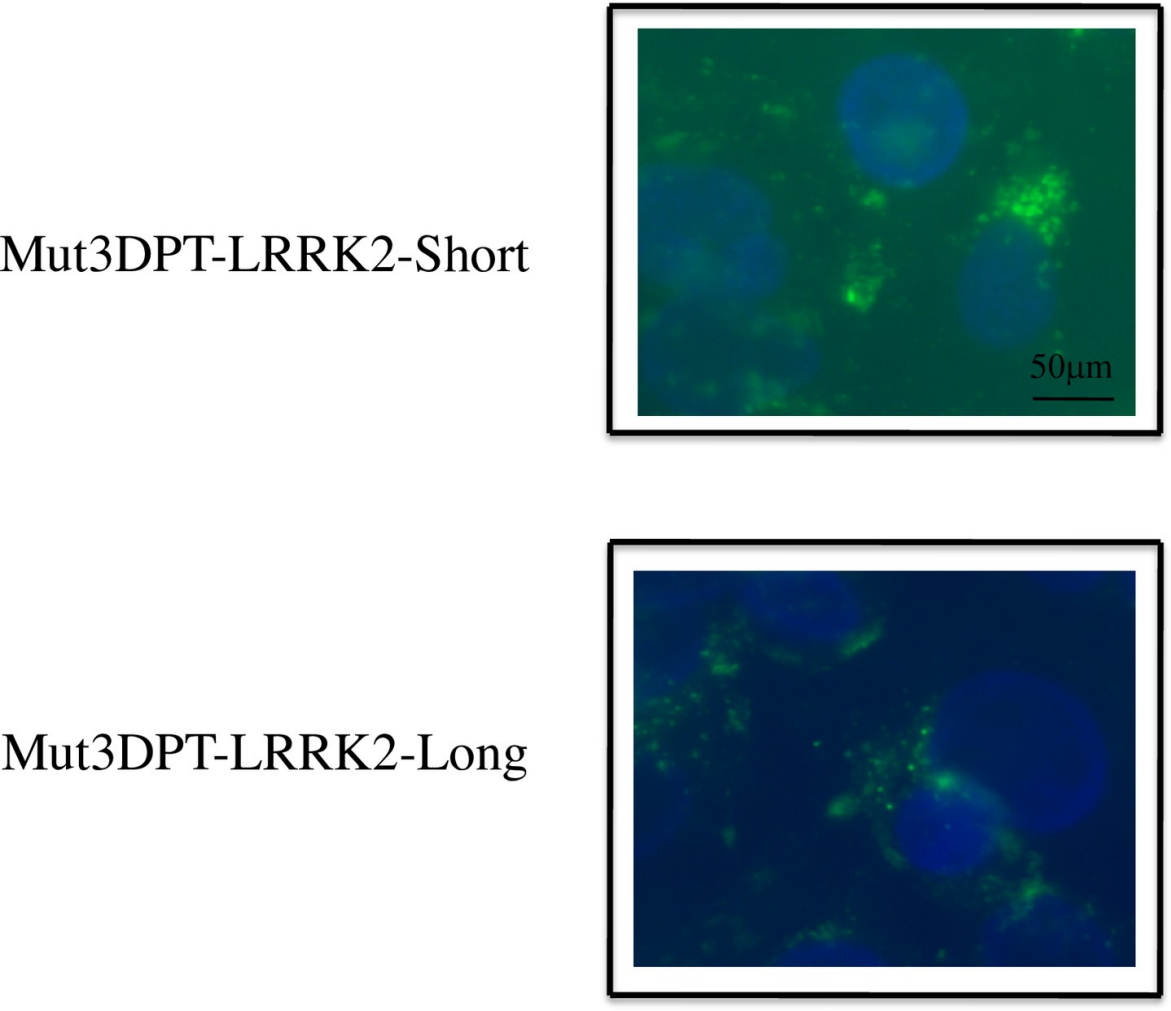
Intracellular localization of FITC-labelled Mut3DPT-LRRK2-Short and Mut3DPT-LRRK2-Long peptides. MDA-MB231 cells were grown on coverslips and incubated 4h at 37°C with 25 μM of FITC-labelled Mut3DPT-LRRK2-Short and Mut3DPT-LRRK2-Long peptides. Cells were washed 3 times with PBS, fixed with 4% paraformaldehyde (PFA) and analysed by fluorescence microscopy (Olympus) using 40x magnification objective. Nuclei were stained with DAPI.

To confirm flow cytometry results and to visualize the intracellular distribution of Mut3DPT-LRRK2-Short and Mut3DPT-LRRK2-Long peptides, MDA-MB231 cells where incubated 4h with a peptide concentration of 25 μM and the distribution of internalized peptide determined by fluorescence microscopy (Fig 7, nucleus stained with DAPI). The staining pattern of both peptides was punctuated, indicating may be possible association with a specific organelle.

### Resistance of the peptide to protease degradation

**Fig 8:**
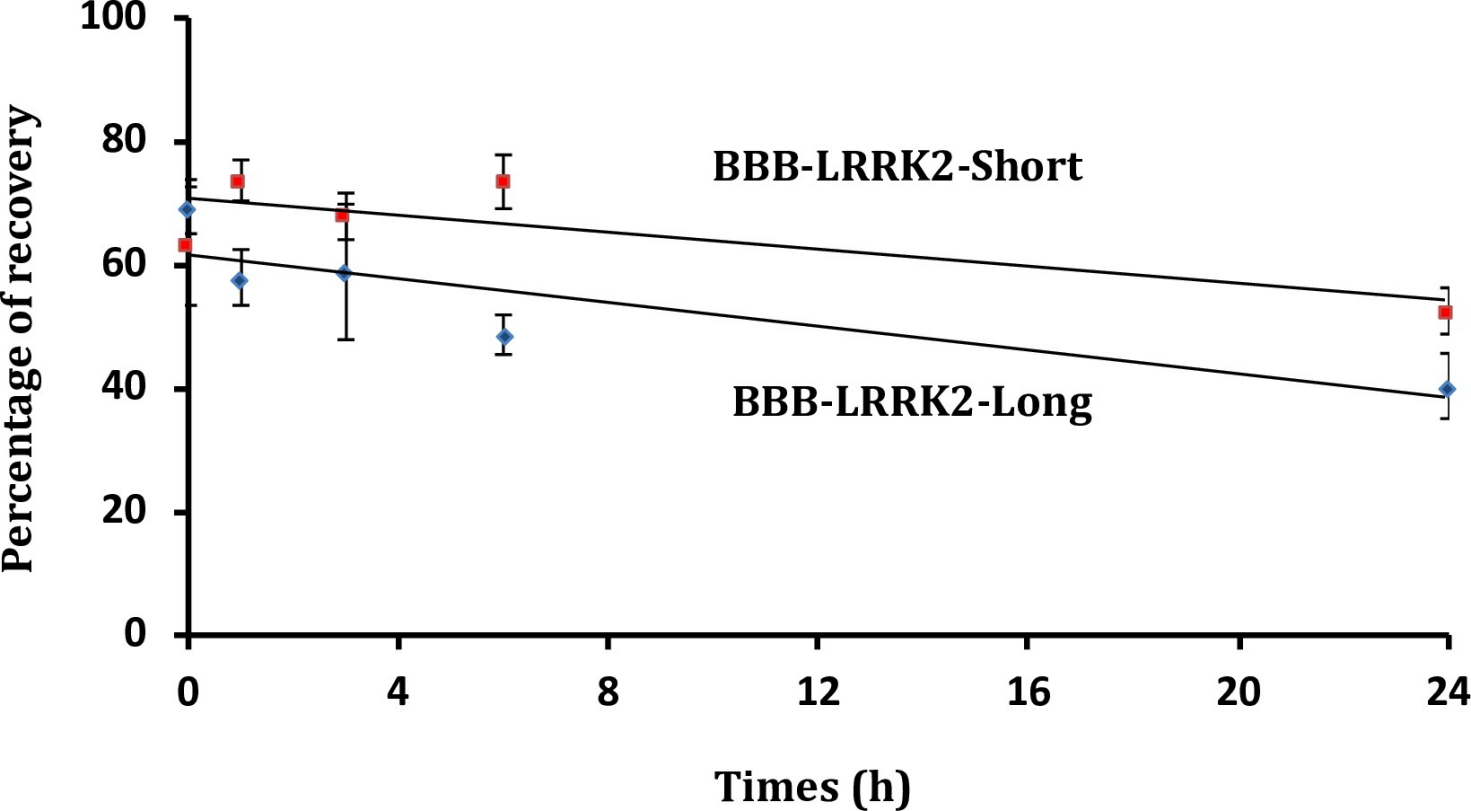
Stability of the new generated peptides BBB-LRRK2-Short and BBB-LRRK2-Long in human serum. Peptides were incubated at 37°C in human serum for different periods of time and their integrity (percentage of intact peptide) was analysed by mass spectrometry. Every measurement was performed in triplicate. Standard deviation is shown.

Given that the key PP1/LRRK2 interaction takes place in the neurons, the interfering peptides were fused to the shuttle THR (THRPPMWSPVWP), able to cross the blood brain barrier (BBB), thus generating two new peptides, BBB-LRRK2-Short and BBB-LRRK2-Long. Their resistance to degradation by proteases present in human serum was determined at 37°C for different periods of time (Fig 8). Both peptides displayed only very limited degradation as detected by mass spectrometry (MS), indicating that they are worthy of further characterization.

### PP1/LRRK2 targeting and cell penetration of BBB-peptides

**Fig 9:**
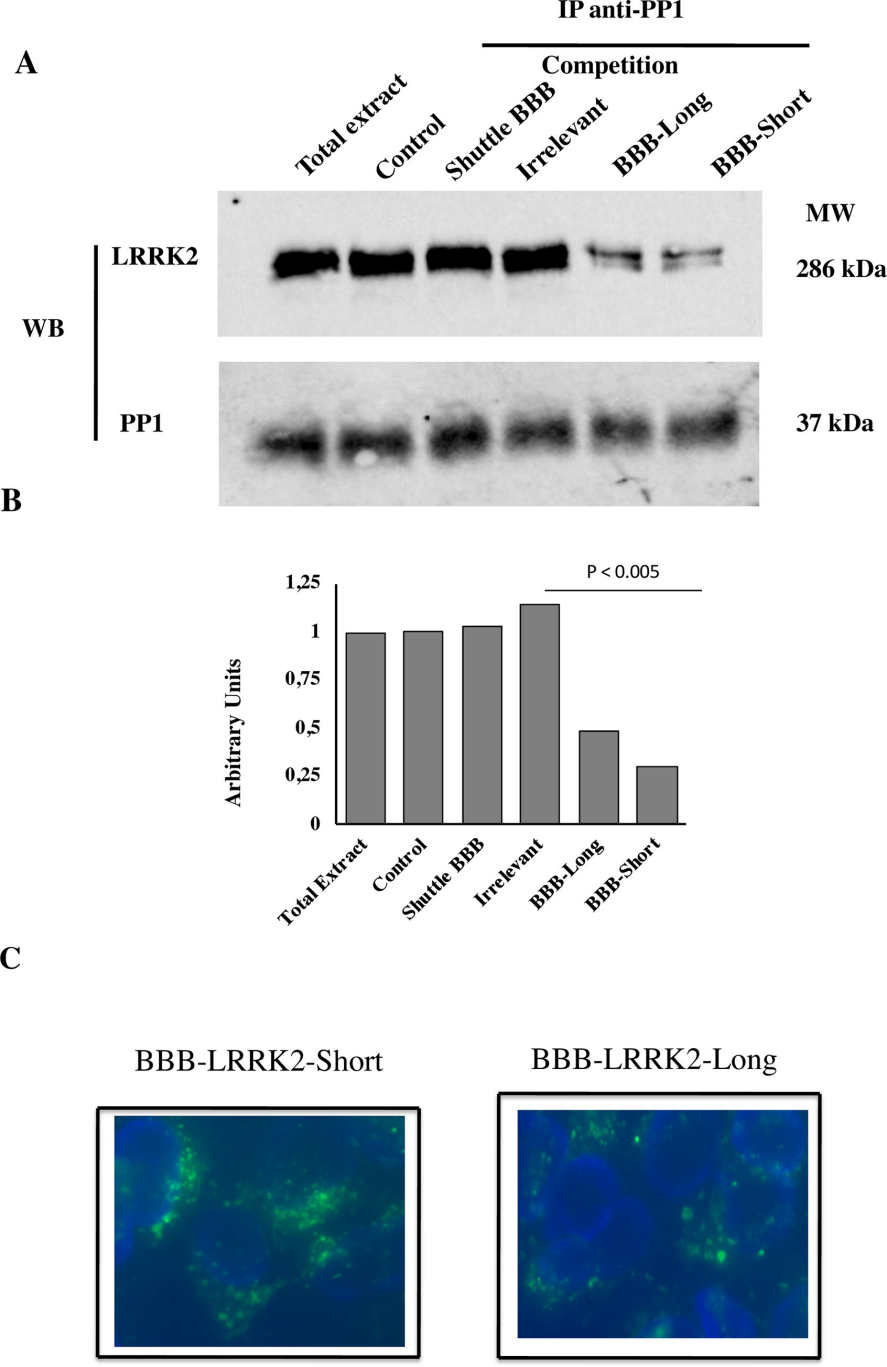
Competition of PP1/LRRK2 interaction and internalization of BBB-peptides. A) MDA-MB231 cells were lysed and immunoprecipitated with anti-PP1 antibody. The interaction PP1/LRRK2 was competed *in vitro* with 1 mM of BBB-Long and BBB-Short peptides. As control, the interaction was competed whit the shuttle alone or an irrelevant peptide. PP1 expression was used as control of loading. Similar result was obtained in two independent experiments. B) Densitometric analysis of the western blot shown statistical analysis. C) MDA-MB 231 cells were grown on coverslip and incubated 4h at 37°C with 25 μM of FITC-labelled BBB-Long and BBB-Short peptides. Cells were washed, fixed and analyzed by fluorescence microscopy (Olympus) as in Fig 7. Magnification 40x. Data are representative of two independent experiments. Nuclei were stained with DAPI.

We further analysed whether these newly generated peptides were able to compete with the PP1/LRRK2 interaction. Lysates from MDA-MB231 cells were immunoprecipitated with an anti-PP1 antibody and *in vitro* binding of LRRK2 competed using 1mM of BBB-Long and BBB-Short peptides (Fig 9A). Both peptides competed with the PP1 binding to LRRK2, in contrast to shuttle alone or an irrelevant peptide. Total extracts, as well as expression of PP1 in all conditions is shown. Fig 9B shows the densitometric analyse of the western blot bands. In addition, we determined whether BBB-Long and BBB-Short peptides were internalized into cells (Fig 9C). MDA-MB231 cells were cultured 4h in the presence of 25 μM of FITC labelled peptides and then analysed by fluorescence microscopy. BBB-Long and BBB-Short peptides penetrated into the cells with a staining pattern similar to that shown in Fig 7. So, independently of the shuttle used, both long and short peptides were able to enter cells and ablate PP1 binding of LRRK2.

## Discussion

Several serine/threonine protein kinase modulators (particularity inhibitors) have been developed and some of them have been considered suitable for further therapeutic development. In parallel, protein-protein interactions subtly(PPI) regulate various aspects of cell homeostasis and deregulation of these interactions are often associated with pathology[29]. For these reasons, we are interested in developing peptides acting as phosphatase modulators by targeting the enzymatic or the partners’ interacting regions. We have developed interfering peptides (IPs) targeting interaction of PP2 with some of its partners[18]. In this study, we targeted the interaction between PP1 and LRRK2, a Parkinson’s disease (PD) associated protein. As PP1 is a hub protein, we decided that it would be best to identify peptides targeting PP1 with LRRK2 derived peptides. The 18 amino acids of LRRK2 (PMGFWSRLINRLLEISPY) that mediate binding of PP1 formed an alpha helix structure, located in an exposed region of the LRRK2 protein. From this sequence and using *in silico* approaches,5 new IPs were synthesized and sequences fused to an optimized penetrating peptide[17] to generate chimeric peptides able to penetrate into cells and ablate PP1 binding to LRRK2. The interfering activity of Mut3DPT-LRRK2-Long and Mut3DPT-LRRK2-Short was confirmed using *in vitro* competition assays. By using Mut3DPT-LRRK2-Long and Mut3DPT-LRRK2-Short peptides fused to a fluorescent marker (FITC), we were able to show that the shorter version of the peptide showed better internalization in both cells lines and in primary cells. The interfering sequence has also been associated to a shuttle that cross the BBB, generating new peptides for use in *in vivo* experiments[30].

Mut3DPT-LRRK2-Long and Mut3DPT-LRRK2-Short peptides performed better PP1/LRRK2 modulation, compared to Mut3DPT-LRRK2-5- to 8. The inactivity of Mut3DPT-LRRK2-5- to 8 suggested that several residues in Mut3DPT-LRRK2-Long are involved in the binding to PP1, or that the structural properties of the half-peptides are not those predicted (off-target and non specific binding could occur), or that they could be less stable than expected, possibly when linked to Mut3DPT.

LRRK2 protein is phosphorylated at multiple sites but the regulation of its phosphorylation is not fully understood[11, 14, 15, 31, 32]. Changes in the phosphorylation status of LRRK2 are linked to the pathogenesis of LRRK2-related PD and the available data show that phosphorylation is a highly regulated physiological event in the disease. It has been shown that PP1 is the phosphatase that efficiently dephosphorylates LRRK2[9], although there is also evidence that, under specific conditions, other serine/threonine phosphatase, such as PP2A, may play an auxiliary role in dephosphorylation of LRRK2. LRRK2 dephosphorylation involves enhanced access of PP1 to LRRK2 phosphosites and that most of the LRRK2 PD mutations have decreased Ser phosphorylation[14, 15, 32]. The cell penetrating and interfering peptides described herein therefore represent important tools to control LRRK2binding to PP1 and probably, phosphorylation of LRRK2. Taken together, they represent new tools to manipulate and to study the PP1/LRRK2 interaction under normal and pathological conditions with the immediate perspective to analyze the potential of these peptides *in vivo* in appropriated mouse models of PD.

## Supporting information

S1 Raw data (PPTX)

S1 Data (PDF)

S2 Data (PDF)

S3 Data (PDF)

S4 Data (PDF)

S5 Data (PDF)

S6 Data (PDF)
