# Supplementary material for: Identification of peptides interfering with the LRRK2/PP1 interaction
Source: PLoS One. 2020 Aug 13;15(8):e0237110. doi: 10.1371/journal.pone.0237110 (PMC7425875; doi:10.1371/journal.pone.0237110)
Supplement: S1 Raw data — (PPTX) [file pone.0237110.s001.pptx]

## Slide 1
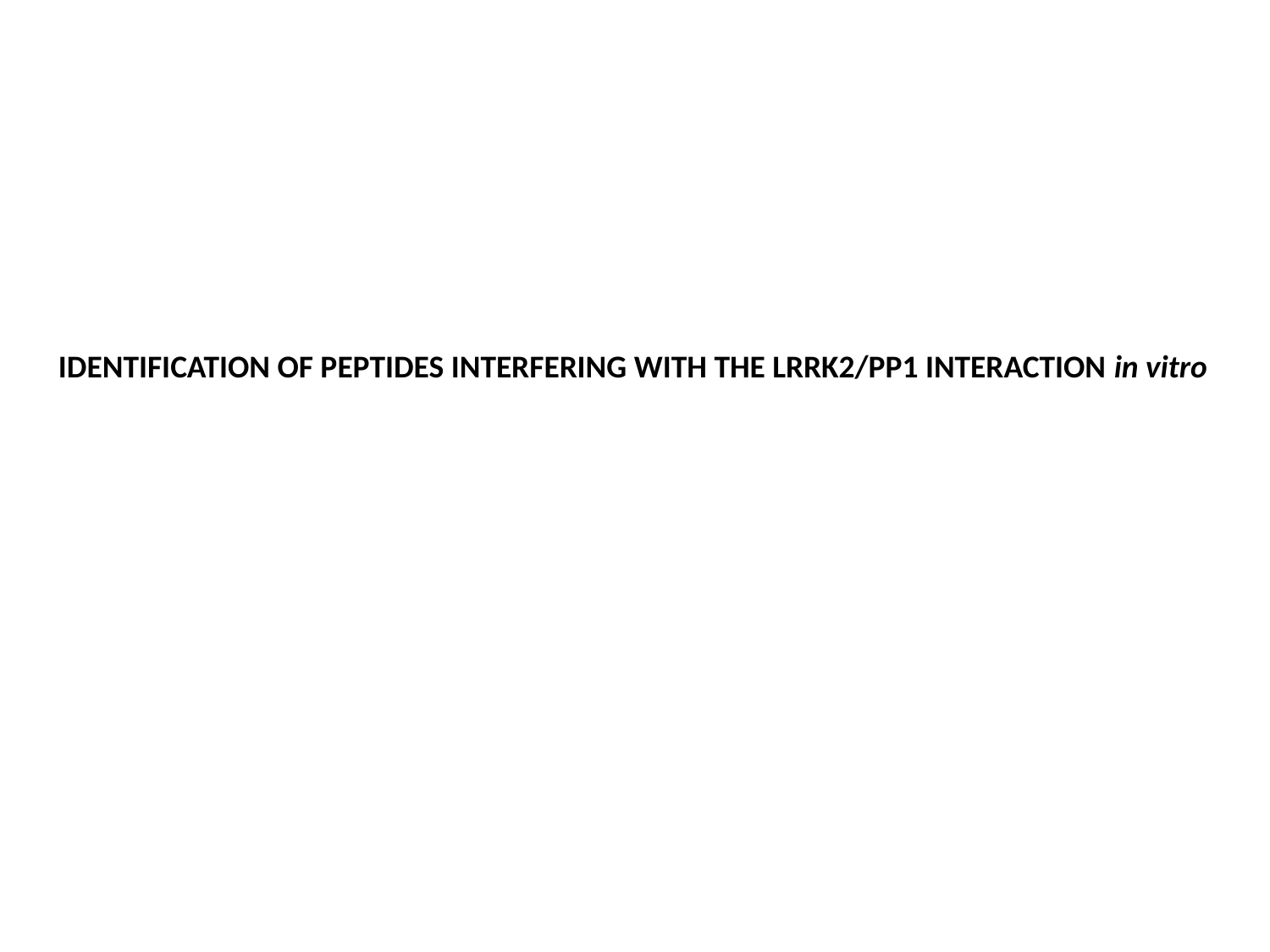

IDENTIFICATION OF PEPTIDES INTERFERING WITH THE LRRK2/PP1 INTERACTION in vitro

## Slide 2
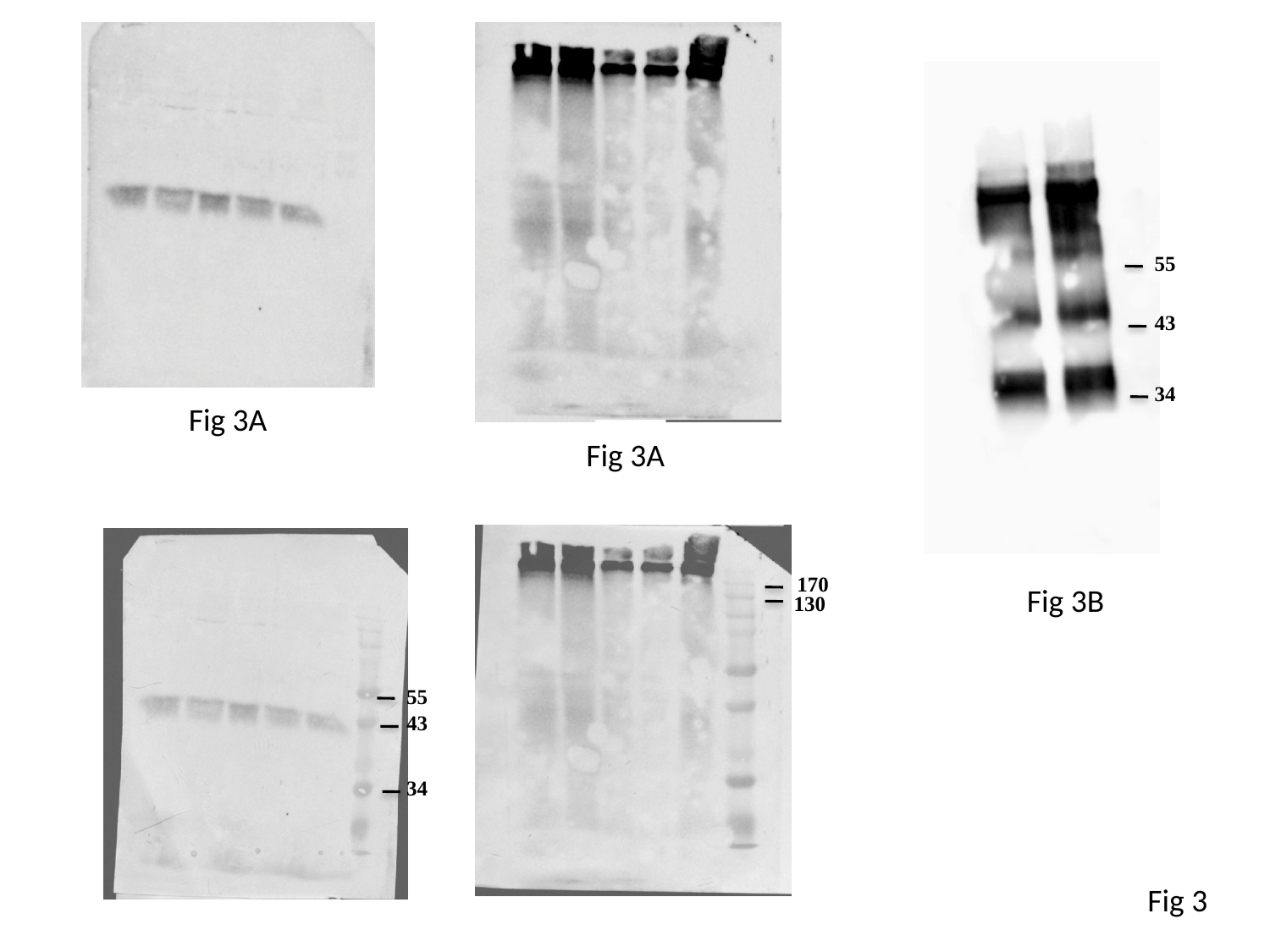

55
43
34
Fig 3A
Fig 3A
170
Fig 3B
130
55
43
34
Fig 3

## Slide 3
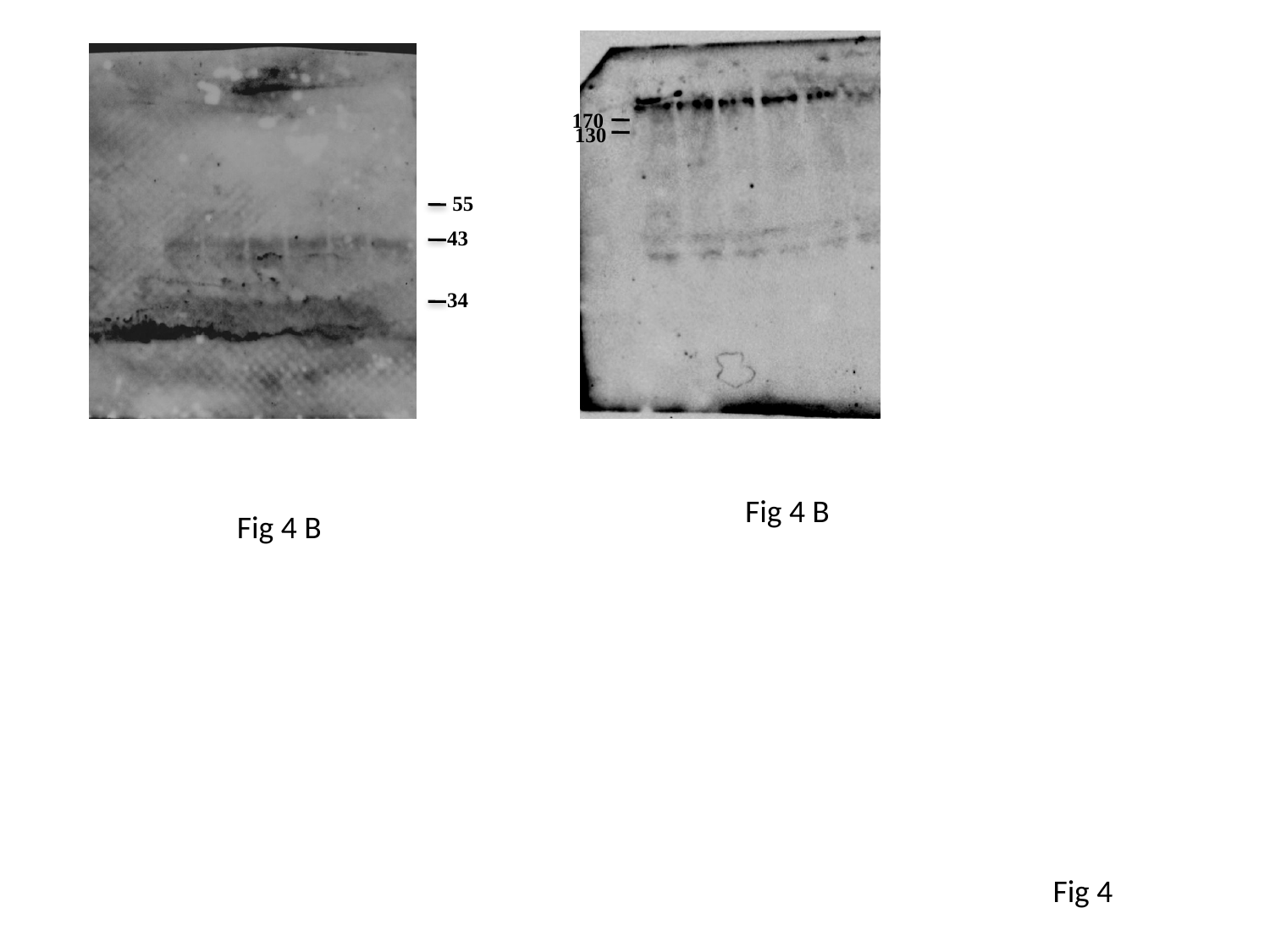

170
130
55
43
34
Fig 4 B
Fig 4 B
Fig 4

## Slide 4
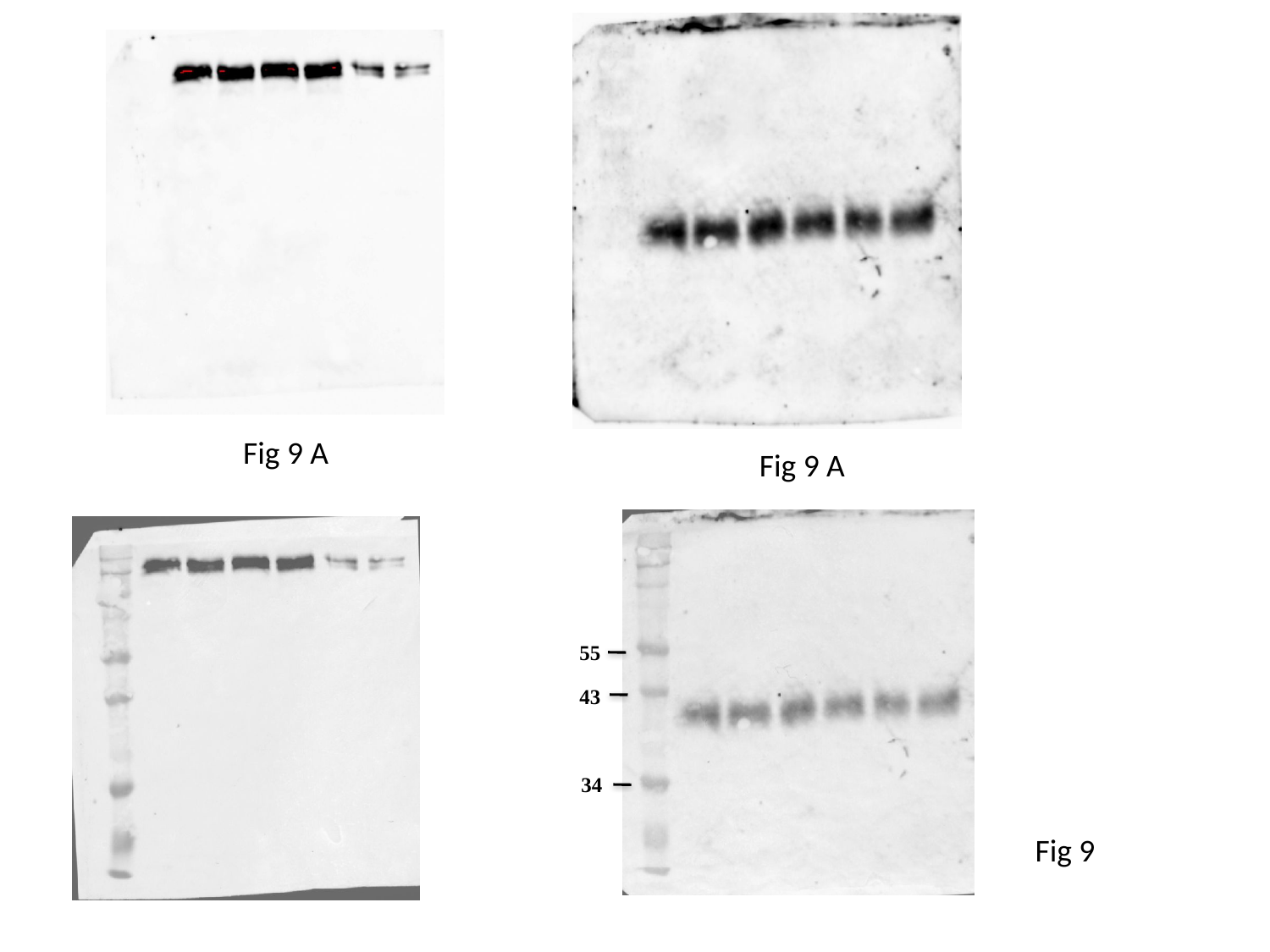

Fig 9 A
Fig 9 A
55
43
34
Fig 9
