# Supplementary figures and images for: Identification of peptides interfering with the LRRK2/PP1 interaction
Source: PLoS One. 2020 Aug 13;15(8):e0237110. doi: 10.1371/journal.pone.0237110 (PMC7425875; doi:10.1371/journal.pone.0237110)

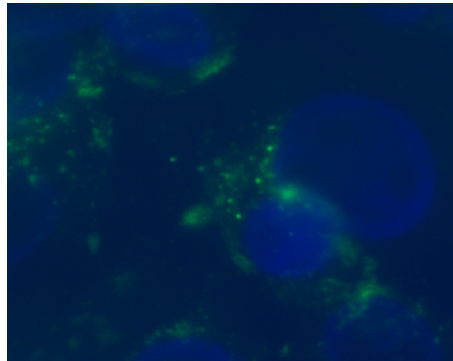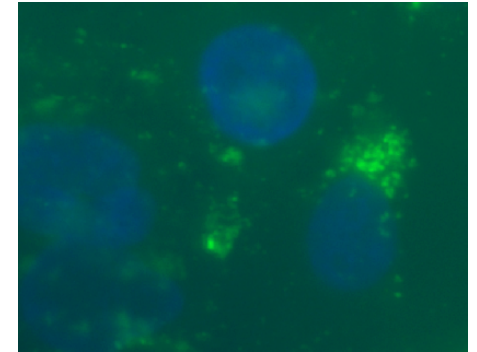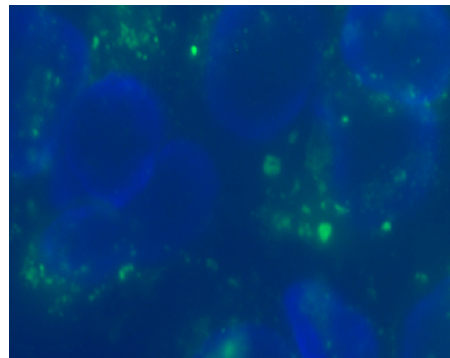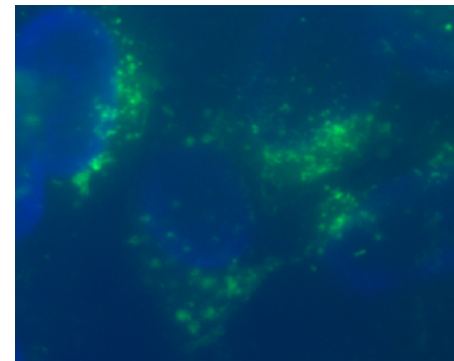

Supplement: S1 Data — (PDF) [file pone.0237110.s002.pdf]
