# Supplementary material for: Identification of peptides interfering with the LRRK2/PP1 interaction
Source: PLoS One. 2020 Aug 13;15(8):e0237110. doi: 10.1371/journal.pone.0237110 (PMC7425875; doi:10.1371/journal.pone.0237110)
Supplement: S2 Data — (PDF) [file pone.0237110.s003.pdf]

FACSDiva Version 6.1.3

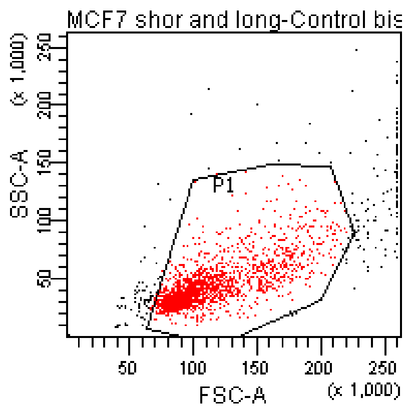

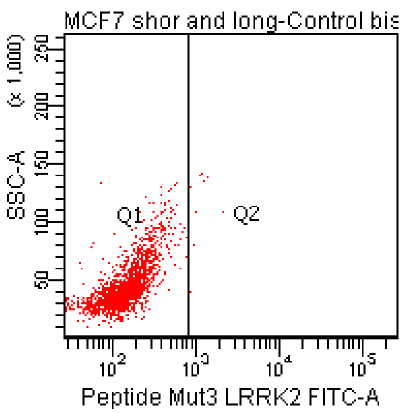

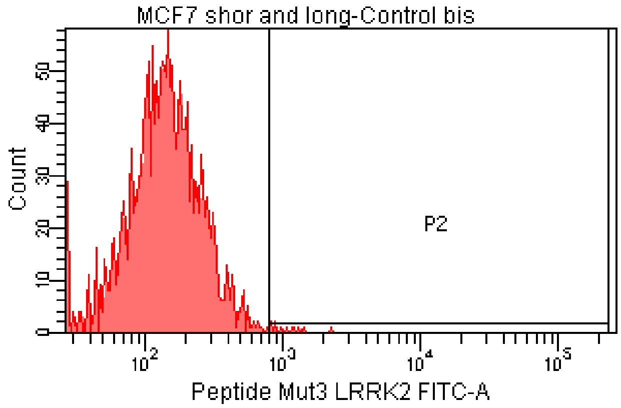

Experiment Name: Peptides FITC
Specimen Name: MCF7 shor and long
Tube Name: Control bis
Record Date: Feb 14, 2018 4:22:47 PM
$OP: Angelita
GUID: ca2fd64e-a749-47be-ba14-2aa0e4e28db8

Population	#Events	%Parent	Peptide Mut3... Mean	Peptide Mut... Median
☒ P2	12	0.6	1,144	1,023
☒ Q2	11	0.6	1,175	1,070

FACSDiva Version 6.1.3

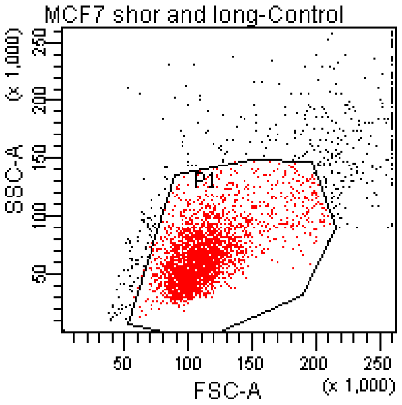

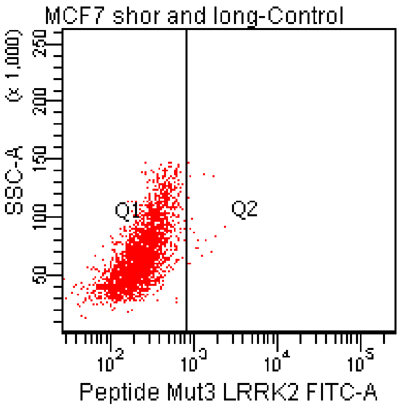

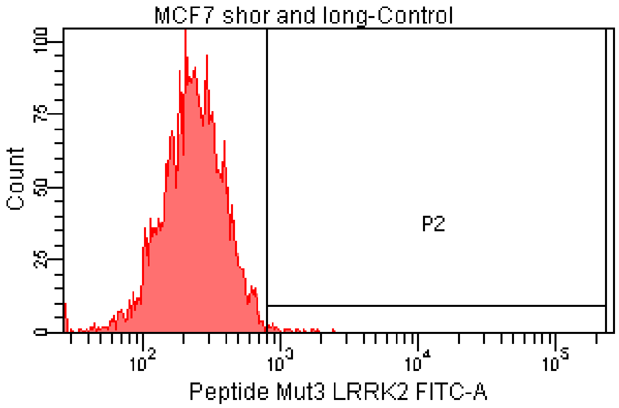

Experiment Name: Peptides FITC
Specimen Name: MCF7 shor and long
Tube Name: Control
Record Date: Feb 14, 2018 4:12:02 PM
$OP: Angelita
GUID: 9e335bdd-0e7b-413a-9735-5b65e4b90ddf

Population	#Events	%Parent	Peptide Mut3... Mean	Peptide Mut... Median
☒ P2	25	0.8	1,148	950
☒ Q2	22	0.7	1,196	1,032

FACSDiva Version 6.1.3

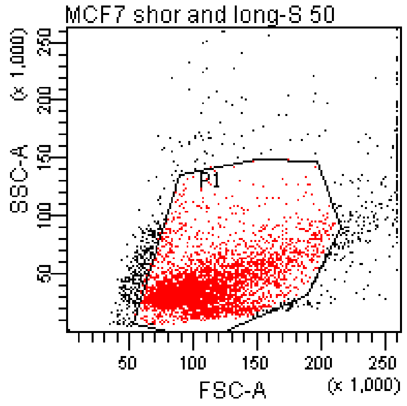

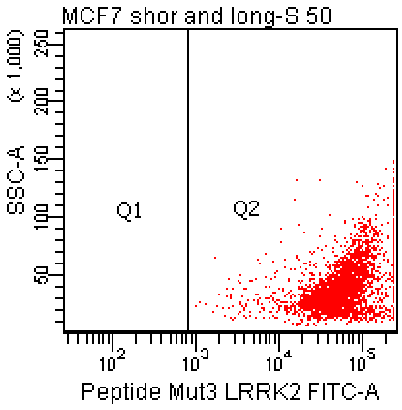

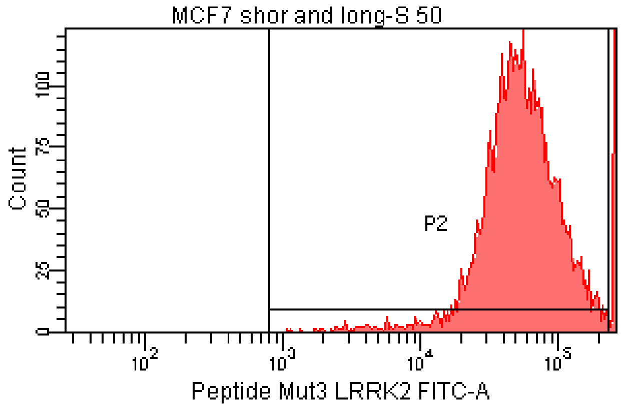

Experiment Name:	Peptides FITC
Specimen Name:	MCF7 shor and long
Tube Name:	S 50
Record Date:	Feb 14, 2018 4:27:22 PM
$OP:	Angelita
GUID:	15c9c5ae-f59a-4e11-9770-2157e94e81e9

Population	#Events	%Parent	Peptide Mut3... Mean	Peptide Mut... Median
☒ P2	4,172	93.5	63,182	53,708
☒ Q2	4,462	100.0	76,032	56,247

FACSDiva Version 6.1.3

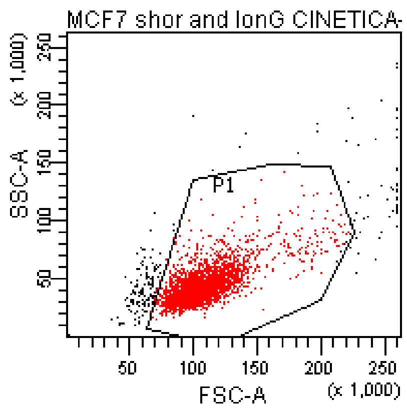

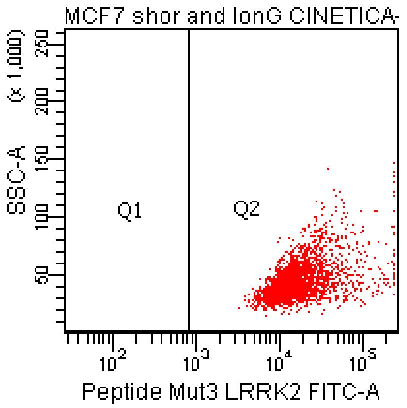

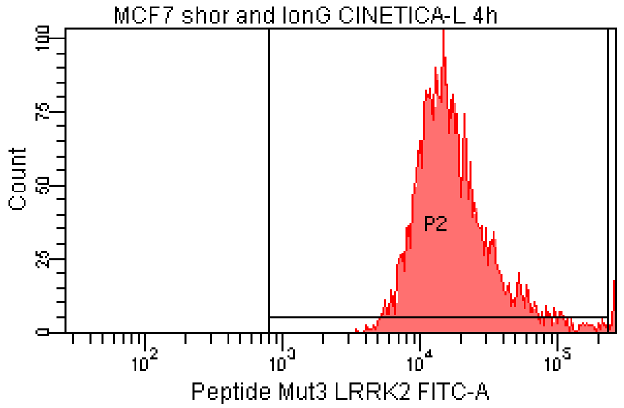

Experiment Name: Peptides FITC
Specimen Name: MCF7 shor and lonG CINETICA
Tube Name: L 4h
Record Date: Feb 15, 2018 12:58:34 PM
$OP: Angelita
GUID: 1722ece6-bbfc-4f19-8e7f-246561d61184

Population	#Events	%Parent	Peptide Mut3... Mean	Peptide Mut... Median
☒ P2	2,972	99.1	22,910	15,993
☒ Q2	3,000	100.0	25,125	16,072

FACSDiva Version 6.1.3

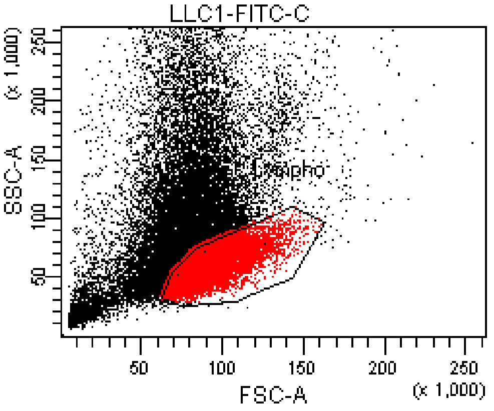

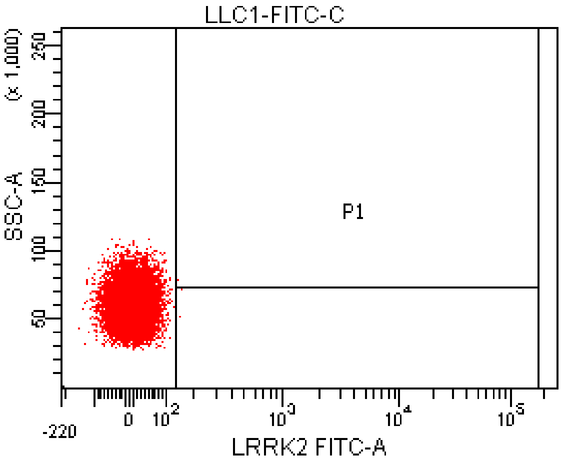

Tube: C

Population	#Events	%Parent	%Total
All Events	29,645	####	100.0
Lympho	10,883	36.7	36.7
P1	3	0.0	0.0
P4	37	0.1	0.1

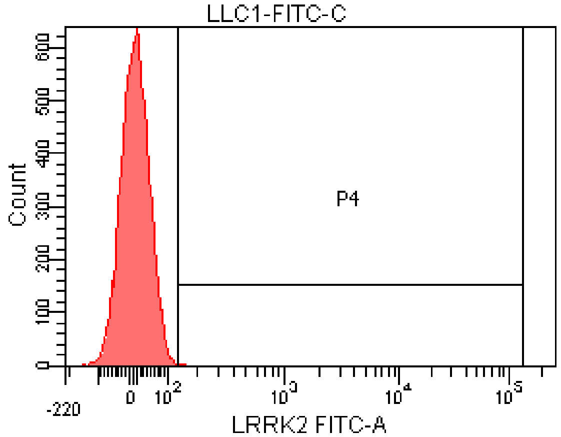

Experiment Name:	PBMC LRRK2 FITC
Specimen Name:	LLC1-FITC
Tube Name:	C
Record Date:	Feb 20, 2018 1:52:38 PM
$OP:	Angelita
GUID:	9d93d456-2a8e-4b65-a7b0-7d64190d3...

Population	#Events	%Parent	LRRK2 FITC-A Mean
All Events	29,645	####	4
Lympho	10,883	36.7	4
P1	3	0.0	147
P4	37	0.1	380

FACSDiva Version 6.1.3

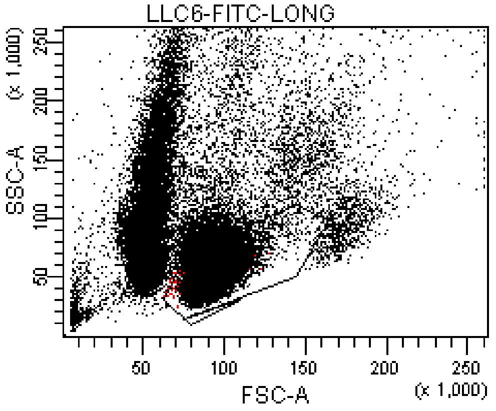

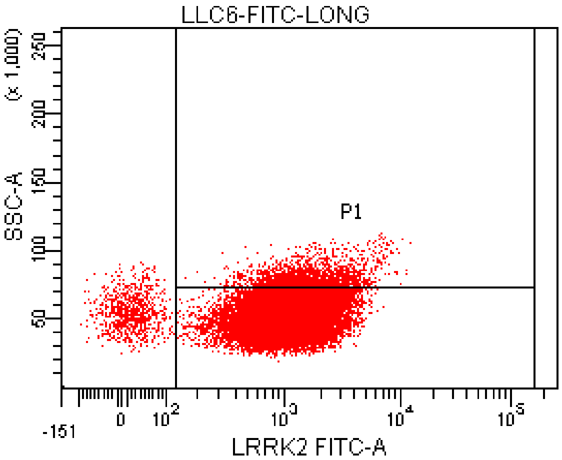

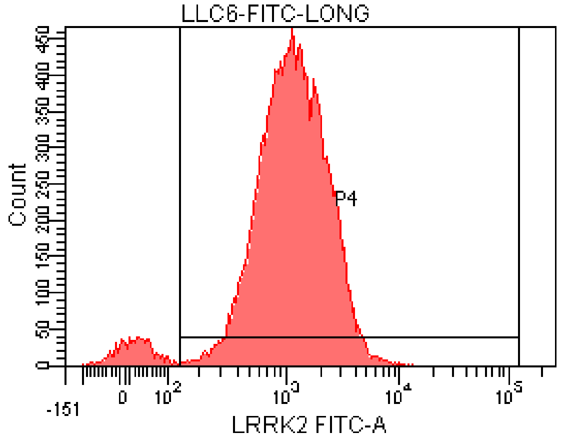

Tube: LONG

Population	#Events	%Parent	%Total
All Events	54,152	####	100.0
Lympho	20,000	36.9	36.9
P1	19,152	95.8	35.4
P4	51,373	94.9	94.9

Experiment Name: PBMC LRRK2 FITC
Specimen Name: LLC6-FITC
Tube Name: LONG
Record Date: Mar 15, 2018 9:43:30 AM
$OP: Angelita
GUID: 28b030dd-aedf-444a-be28-1efd7b6a3c75

Population	#Events	%Parent	LRRK2 FITC-A Mean
All Events	54,152	####	5,485
Lympho	20,000	36.9	1,322
P1	19,152	95.8	1,380
P4	51,373	94.9	5,721

FACSDiva Version 6.1.3

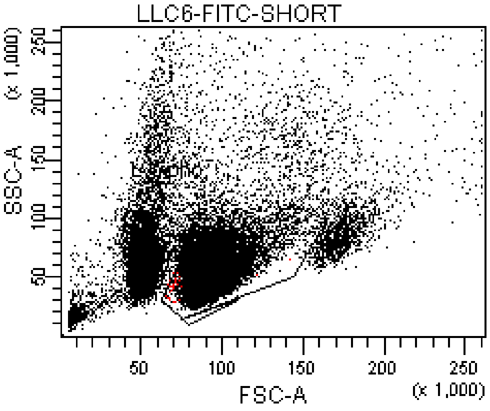

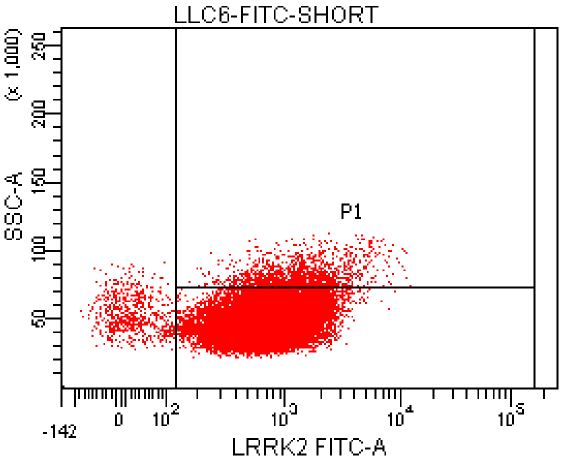

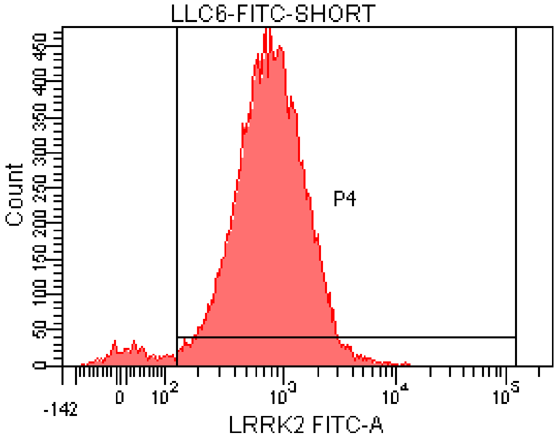

Tube: SHORT

Population	#Events	%Parent	%Total
All Events	36,442	####	100.0
Lympho	20,000	54.9	54.9
P1	19,301	96.5	53.0
P4	34,188	93.8	93.8

Experiment Name: PBMC LRRK2 FITC
Specimen Name: LLC6-FITC
Tube Name: SHORT
Record Date: Mar 15, 2018 9:42:37 AM
$OP: Angelita
GUID: 2cca8865-b91b-4313-855c-b0b477a05821

Population	#Events	%Parent	LRRK2 FITC-A Mean
All Events	36,442	####	2,507
Lympho	20,000	54.9	919
P1	19,301	96.5	952
P4	34,188	93.8	2,646

FACSDiva Version 6.1.3

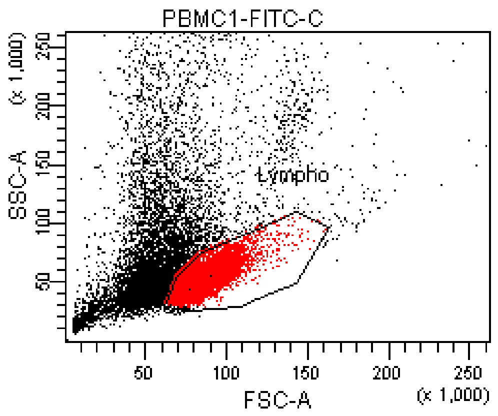

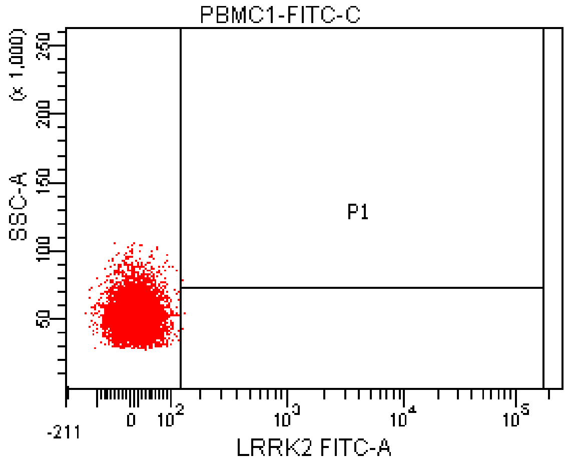

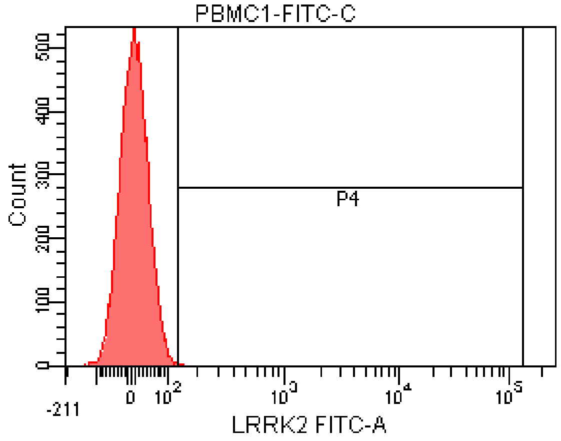

Tube: C

Population	#Events	%Parent	%Total
All Events	16,844	####	100.0
Lympho	9,076	53.9	53.9
P1	3	0.0	0.0
P4	7	0.0	0.0

Experiment Name: PBMC LRRK2 FITC
Specimen Name: PBMC1-FITC
Tube Name: C
Record Date: Feb 20, 2018 1:48:02 PM
$OP: Angelita
GUID: 0bc32205-fbf8-48fd-9a93-dfd756bec3e9

Population	#Events	%Parent	LRRK2 FITC-A Mean
All Events	16,844	####	3
Lympho	9,076	53.9	3
P1	3	0.0	141
P4	7	0.0	173

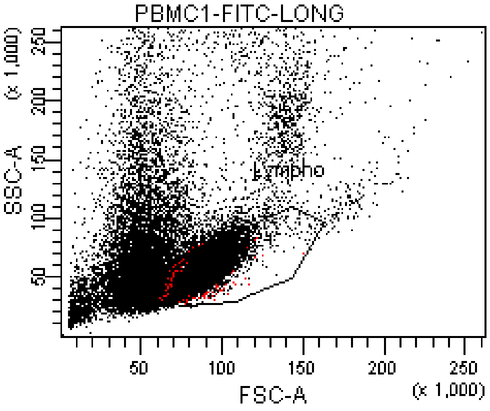

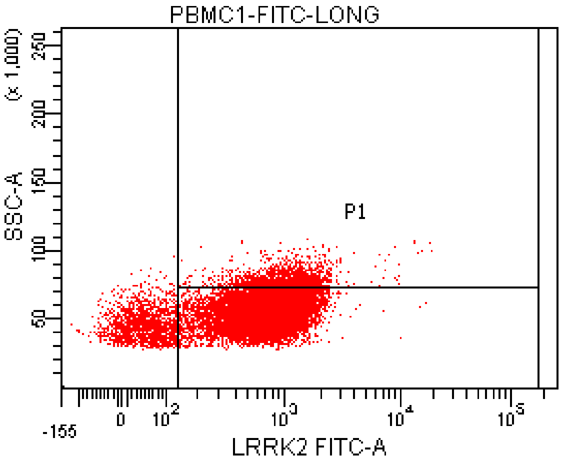

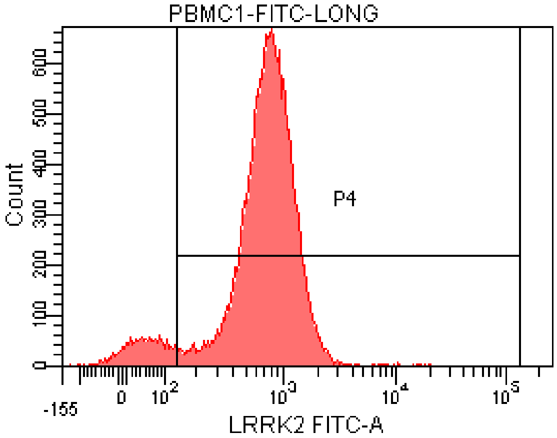

Tube: LONG

Population	#Events	%Parent	%Total
All Events	34,095	####	100.0
Lympho	20,533	60.2	60.2
P1	19,014	92.6	55.8
P4	23,978	70.3	70.3

Experiment Name: PBMC LRRK2 FITC
Specimen Name: PBMC1-FITC
Tube Name: LONG
Record Date: Feb 20, 2018 1:50:35 PM
$OP: Angelita
GUID: 719d85fe-d54b-4bc0-bb8b-67f85e7d681f

Population	#Events	%Parent	LRRK2 FITC-A Mean
All Events	34,095	####	1,655
Lympho	20,533	60.2	780
P1	19,014	92.6	839
P4	23,978	70.3	2,342

FACSDiva Version 6.1.3

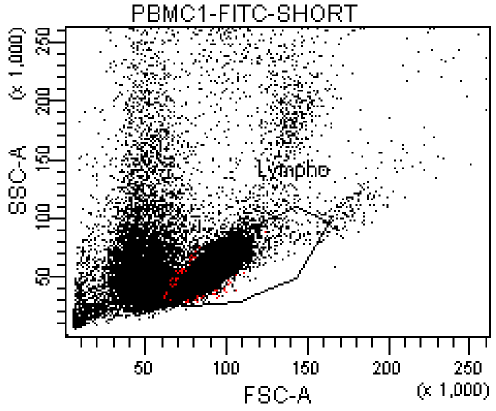

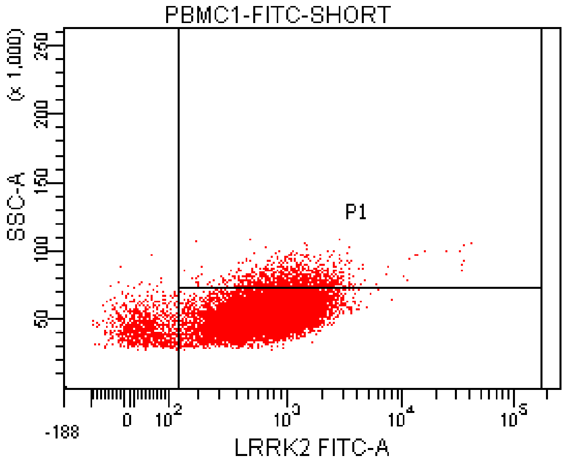

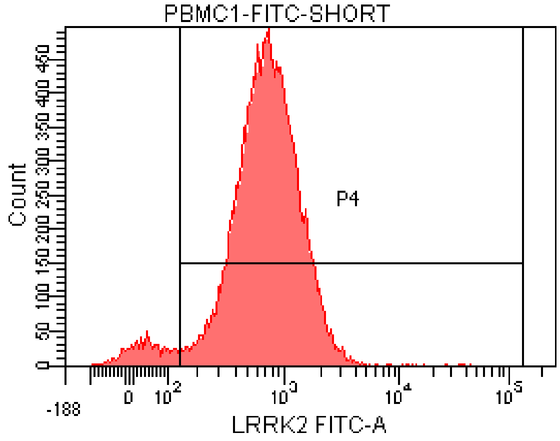

Tube: SHORT

Population	#Events	%Parent	%Total
All Events	30,480	####	100.0
Lympho	18,821	61.7	61.7
P1	17,930	95.3	58.8
P4	22,884	75.1	75.1

Experiment Name: PBMC LRRK2 FITC
Specimen Name: PBMC1-FITC
Tube Name: SHORT
Record Date: Feb 20, 2018 1:49:18 PM
$OP: Angelita
GUID: 219efa85-a0a0-45a1-8adf-259247ed47...

Population	#Events	%Parent	LRRK2 FITC-A Mean
All Events	30,480	####	3,827
Lympho	18,821	61.7	814
P1	17,930	95.3	853
P4	22,884	75.1	5,016
