# Supplementary material for: Identification of peptides interfering with the LRRK2/PP1 interaction
Source: PLoS One. 2020 Aug 13;15(8):e0237110. doi: 10.1371/journal.pone.0237110 (PMC7425875; doi:10.1371/journal.pone.0237110)
Supplement: S3 Data — (PDF) [file pone.0237110.s004.pdf]

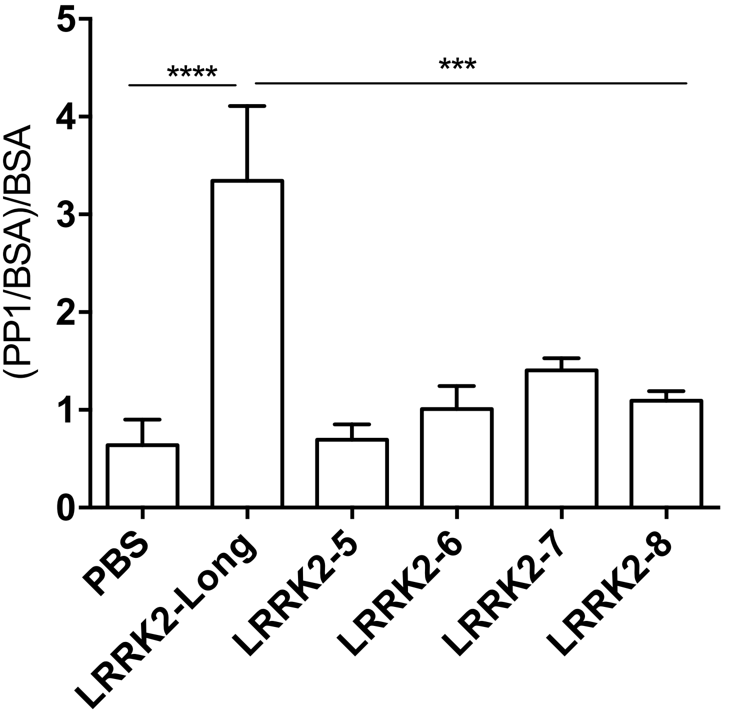

*** P<0.001
**** p<0.0001

Ou comme ça plus simple

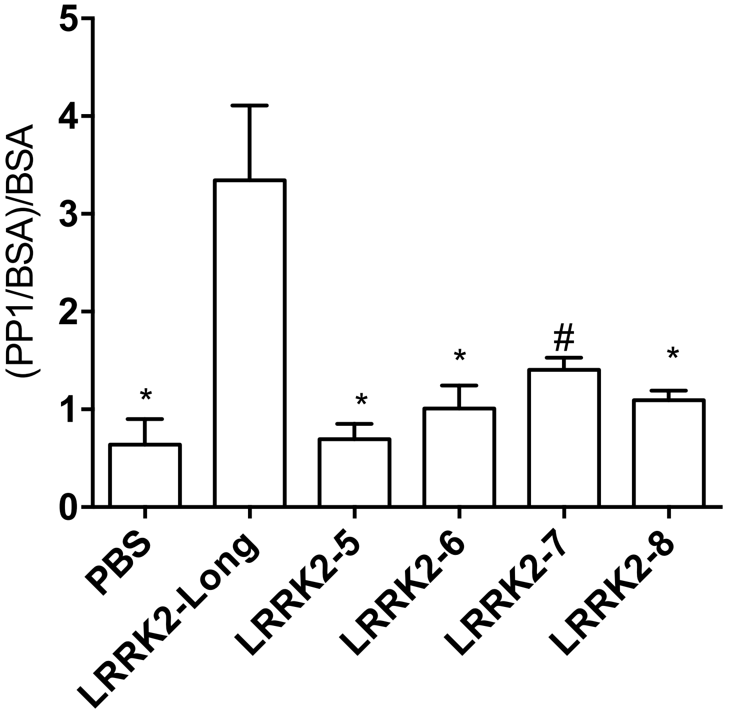

* p<0.0001 vs LRRK-2-Long
# p<0.001 vs LRRK-2-Long

One way Anova

Number of families	1
Number of comparisons per family	5
Alpha	0,05

Dunnett's multiple comparisons test	Mean Diff,	95% CI of diff,	Significant?	Summary		B-?	
LRRK2-Long vs. PBS	2,703	1,861 to 3,545	Yes	****		A	PBS
LRRK2-Long vs. LRRK2-5	2,650	1,808 to 3,492	Yes	****		C	LRRK2-5
LRRK2-Long vs. LRRK2-6	2,333	1,491 to 3,175	Yes	****		D	LRRK2-6
LRRK2-Long vs. LRRK2-7	1,940	1,098 to 2,782	Yes	***		E	LRRK2-7
LRRK2-Long vs. LRRK2-8	2,250	1,408 to 3,092	Yes	****		F	LRRK2-8

Test details	Mean 1	Mean 2	Mean Diff,	SE of diff,	n1	n2	q
LRRK2-Long vs. PBS	3,343	0,6407	2,703	0,2903	3	3	9,311
LRRK2-Long vs. LRRK2-5	3,343	0,6933	2,650	0,2903	3	3	9,130
LRRK2-Long vs. LRRK2-6	3,343	1,010	2,333	0,2903	3	3	8,039
LRRK2-Long vs. LRRK2-7	3,343	1,403	1,940	0,2903	3	3	6,684
LRRK2-Long vs. LRRK2-8	3,343	1,093	2,250	0,2903	3	3	7,752

21/02 exp1

	RLL		PBS	
	A:Y1	A:Y2	A:Y1	A:Y2
PP1-2ug/mL	1.360	1.118	0.244	0.292

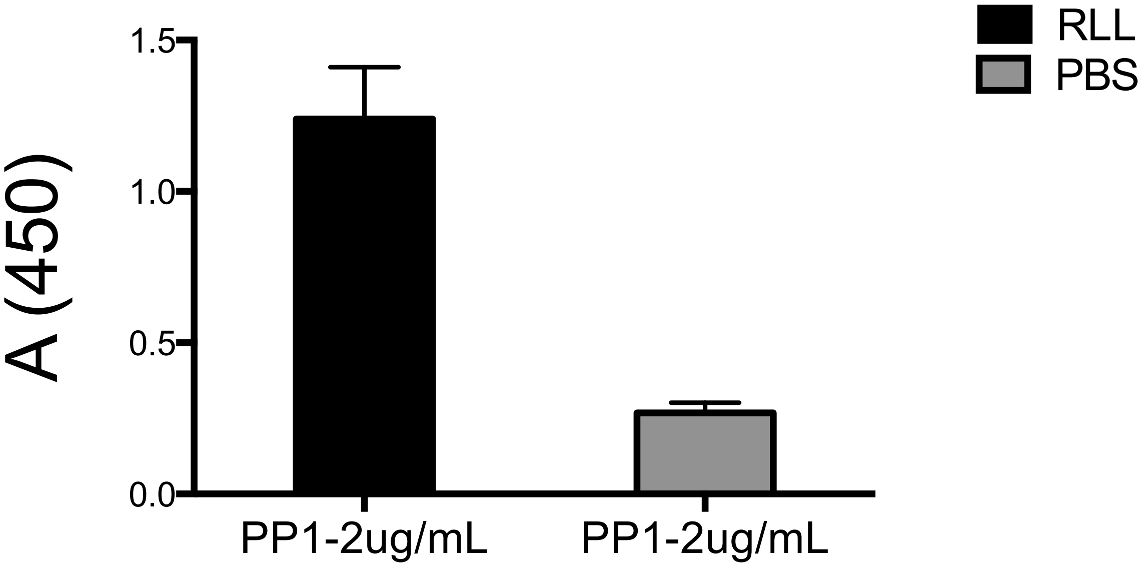

26/02 exp2

	RLL		Ctrl	
	A:Y1	A:Y2	A:Y1	A:Y2
PP1- 2ug/mL	0.353	0.335	0.097	0.096
PP1- 0.6ug/mL	0.067	0.074	0.042	0.041

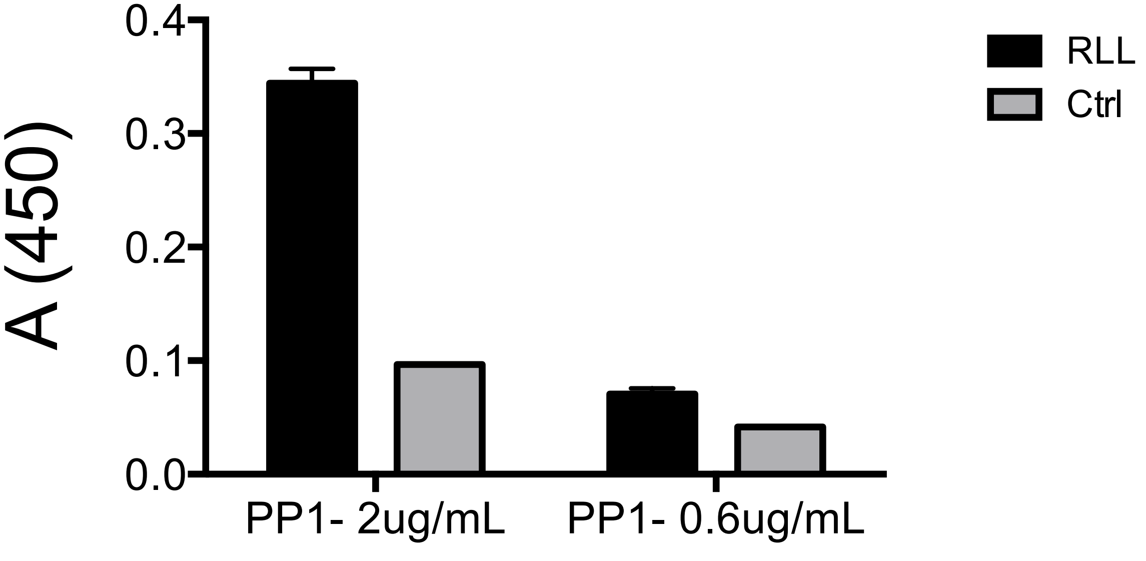

```
"Table Analyzed" "Data 1"

"Column B" CtrL
vs.   vs.
"Column A" PP1

"Unpaired t test"
"  P value" "< 0.0001"
"  P value summary"    ****
"  Significantly different? (P < 0.05)" Yes
"  One- or two-tailed P value?"   Two-tailed
"  t, df"  "t=47.56 df=4"

"How big is the difference?"
"  Mean ¬± SEM of column A" "0.3440 ¬± 0.005196, n=3"
"  Mean ¬± SEM of column B" "0.0965 ¬± 0.0002887, n=3"
"  Difference between means" "-0.2475 ¬± 0.005204"
"  95% confidence interval" "-0.2619 to -0.2331"
"  R squared"    0,9982

"F test to compare variances"
"  F,DFn, Dfd"   "324.0, 2, 2"
"  P value" 0,0062
"  P value summary"    **
"  Significantly different? (P < 0.05)" Yes
```
