# Supplementary material for: Identification of peptides interfering with the LRRK2/PP1 interaction
Source: PLoS One. 2020 Aug 13;15(8):e0237110. doi: 10.1371/journal.pone.0237110 (PMC7425875; doi:10.1371/journal.pone.0237110)

Mut3DPT-LRRK2-Short
Mean Fluorescence
Peptide Concentration (µM)

	0	10	25	50
	980	25100	44890	67000
	1090	25950	45100	66950
	1110	27100	47220	69300
Average	**1060**	**26050**	**45736,667**	**67750**
SD	70	1003,743	1288,8884	1342,5722
Error	40,414519	579,51129	744,14007	775,1344

Mut3DPT-LRRK2-Long
Mean Fluorescence
Peptide Concentration (µM)

0	10	25	50
980	14110	30010	38120
1090	14780	30270	38890
1110	14900	30980	39900
**1060**	**14596,667**	**30420**	**38970**
70	425,71508	502,09561	892,69256
40,414519	245,78672	289,88503	515,39629

p values comparison short vs long

Peptide Concentration (µM)

10	25	50
0.00005	0.00004	0.00001

Mut3DPT-LRRK2-Short
Mean Fluorescence
Time (h)

	0	0,08	0,5	1	2	3	4
	950	6300	8400	20290	23810	35300	37490
	1120	6987	8590	20890	24200	35600	38000
	1220	7190	8910	21150	25000	37090	38720
Average	**1096,6667**	**6825,6667**	**8633,3333**	**20776,667**	**24336,667**	**35996,667**	**38070**
SD	136,50397	466,41862	257,74665	441,05933	606,65751	958,66226	617,98058
Error	78,810603	269,28692	148,8101	254,64572	350,25388	553,48392	356,79126

Mut3DPT-LRRK2-Long
Mean Fluorescence
Time (h)

0	0,08	0,5	1	2	3	4
1000	3420	7890	9050	16900	22980	28800
1250	3780	8450	9220	17210	23300	29130
1380	3990	9010	9780	18140	25100	32140
**1210**	**3730**	**8450**	**9350**	**17416,667**	**23793,333**	**30023,333**
193,13208	288,27071	560	381,96859	645,31646	1142,8619	1840,4981
111,50486	166,43317	323,31615	220,52967	372,57363	659,83163	1062,6121

p values comparison short vs long

Time (h)

0	0,08	0,5
0.4532	0.00061	0.63364

1	2	3	4
0	0.00017	0.00014	0.00199

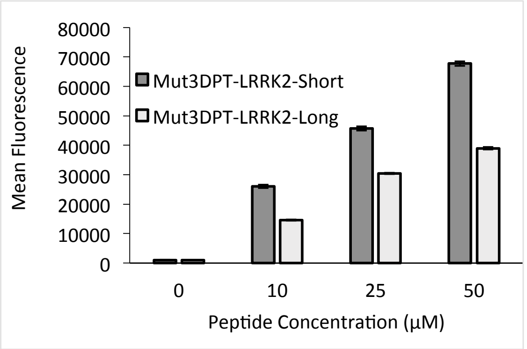

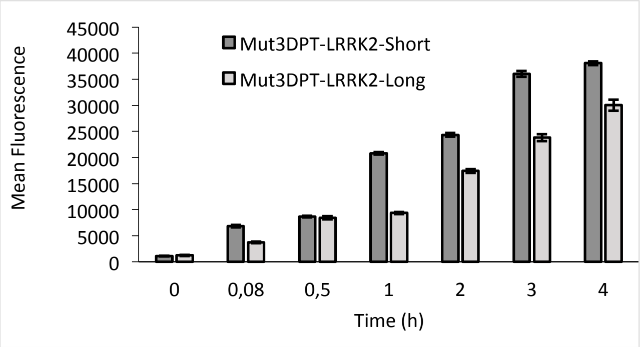

Supplement: S4 Data — (PDF) [file pone.0237110.s005.pdf]
