# Supplementary material for: Identification of peptides interfering with the LRRK2/PP1 interaction
Source: PLoS One. 2020 Aug 13;15(8):e0237110. doi: 10.1371/journal.pone.0237110 (PMC7425875; doi:10.1371/journal.pone.0237110)
Supplement: S5 Data — (PDF) [file pone.0237110.s006.pdf]

0	1	3	6	24
69,43	57,96	58,86	48,69	40,37
63,04	73,68	67,86	73,46	52,53
4,38	4,52	10,98	3,21	5,29
9,6	3,31	3,77	4,35	3,74

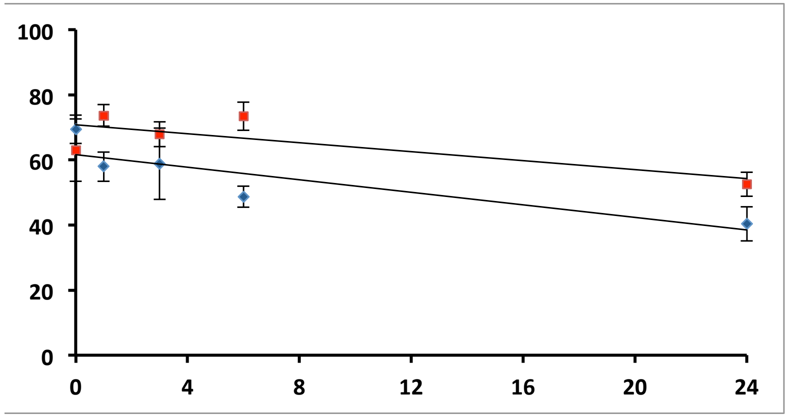

m/z

Temps (H)	Intensité (UA)		Stand. Deviation Intensite	
	peptide n°3 m/z: 3663,64 Da	peptide n°3 m/z: 3087,16 Da	peptide n°3 m/z: 3663,64 Da	peptide n°3 m/z: 3087,16 Da
0	69,43	63,04	4,38	9,6
1	57,96	73,68	4,52	3,31
3	58,86	67,86	10,98	3,77
6	48,69	73,46	3,21	4,35
24	40,37	52,53	5,29	3,74
