# Supplementary material for: Identification of peptides interfering with the LRRK2/PP1 interaction
Source: PLoS One. 2020 Aug 13;15(8):e0237110. doi: 10.1371/journal.pone.0237110 (PMC7425875; doi:10.1371/journal.pone.0237110)

Control	11968.660
Shuttle	10972.075
LRRK2-Long	12804.439
LRRK2-Short	10980.439
Irrelevant	9060.054

21153,823	11968,66	1,767434533	1
18122,116	10972,075	1,65165805	0,934494613
8292,187	12804,439	0,647602523	0,36640821
8740,874	10980,439	0,796040486	0,450393195
15951,953	9060,054	1,76069072	0,996184406

Control	1
Shuttle	0,934494613
LRRK2-Long	0,36640821
LRRK2-Short	0,450393195
Irrelevant	0,996184406

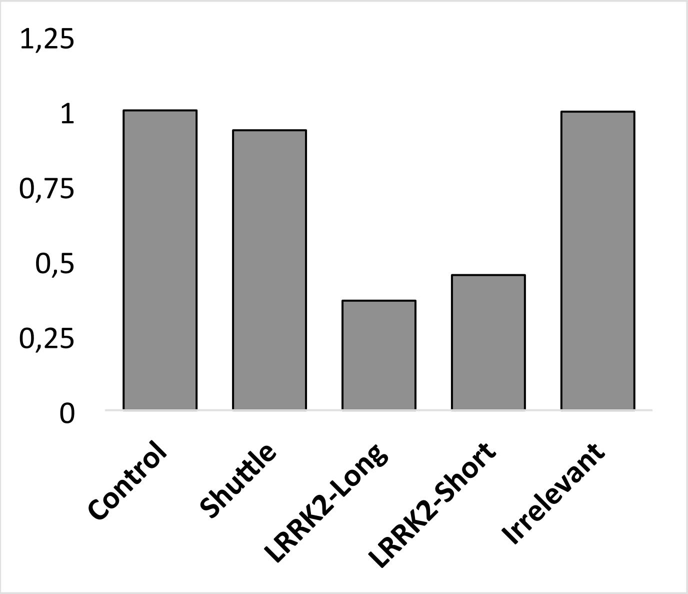

Total Extract	15255.602
Control	14770.238
Shuttle BBB	15976.723
Irrelevant	13611.359
BBB-Long	11717.874
BBB-Short	13257.945

13350,125	15255,602	0,875096571	0,993914932
13004,518	14770,238	0,880454194	1
13635,054	15976,723	0,853432459	1,025384682
13680,518	13611,359	1,005080977	1,141548286
5037,104	11717,874	0,429865008	0,488230973
3535,205	13257,945	0,266648036	0,302852821

Total Extract	0,993914932
Control	1
Shuttle BBB	1,025384682
Irrelevant	1,141548286
BBB-Long	0,488230973
BBB-Short	0,302852821

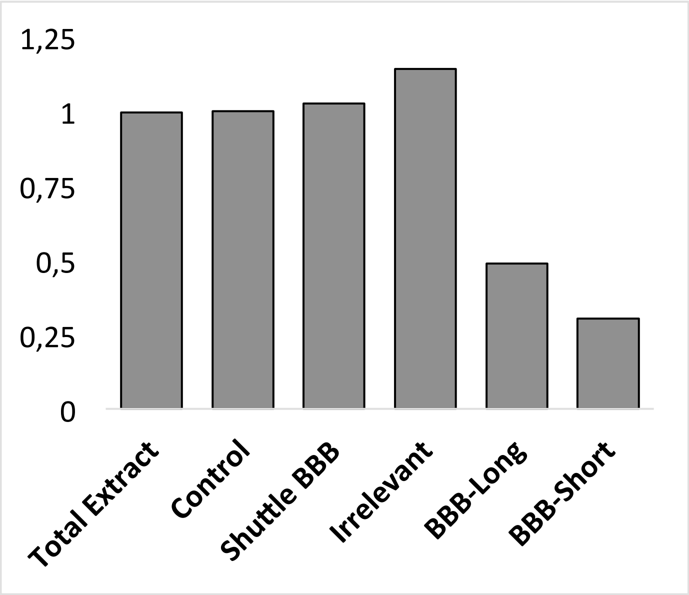

Control	3223.296
LRRK2-7	5070.489
LRRK2-8	8496.380
LRRK2-6	6108.652
LRRK2-5	4184.045
LRRK2-Long	4813.782

7117,296	3223,296	2,208080176	1
6212,125	5070,489	1,225153038	0,554849888
5308,347	8496,38	0,624777493	0,282950547
4404,912	6108,652	0,72109395	0,326570547
2416,891	4184,045	0,5776446	0,261604903
1287,255	4813,782	0,267410323	0,12110535

Control	1
LRRK2-7	0,6565
LRRK2-8	0,5305
LRRK2-6	0,4809
LRRK2-5	0,487
LRRK2-Long	0,12110535

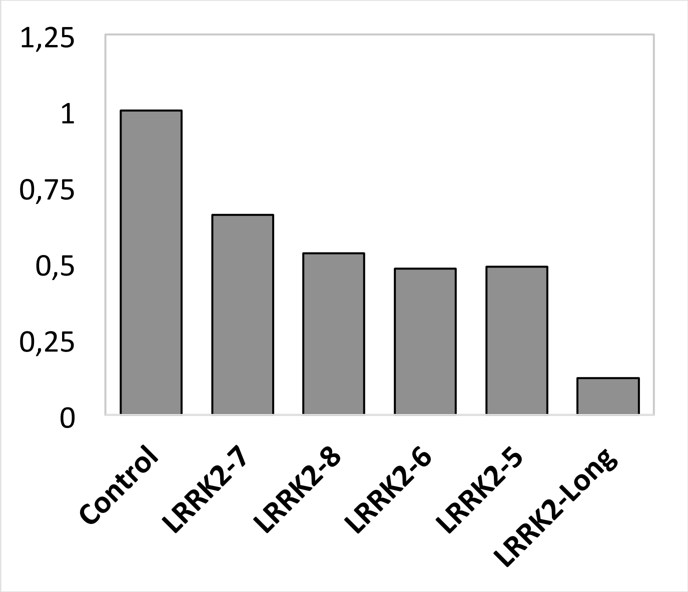

Supplement: S6 Data — (PDF) [file pone.0237110.s007.pdf]
